# Enhanced detection of expanded repeat mRNA foci with hybridization chain reaction

**DOI:** 10.1186/s40478-021-01169-8

**Published:** 2021-04-23

**Authors:** M. Rebecca Glineburg, Yuan Zhang, Amy Krans, Elizabeth M. Tank, Sami J. Barmada, Peter K. Todd

**Affiliations:** 1 Department of Neurology, University of Michigan, 109 Zina Pitcher Place, Ann Arbor, MI 4005 BSRB48109-2200 USA; 2 Department of Respiratory Medicine, Xiangya Hospital, Central South University, Changsha, 410008 China; 3 Veterans Affairs Medical Center, Ann Arbor, MI USA

**Keywords:** *C9orf72*, Amyotrophic lateral sclerosis, Fragile X, FXTAS, Repeat-associated non-AUG (RAN) translation, RNA Gelation, RNA foci, Stress granules, G3BP

## Abstract

Transcribed nucleotide repeat expansions form detectable RNA foci in patient cells that contribute to disease pathogenesis. The most widely used method for detecting RNA foci, fluorescence in situ hybridization (FISH), is powerful but can suffer from issues related to signal above background. Here we developed a repeat-specific form of hybridization chain reaction (R-HCR) as an alternative method for detection of repeat RNA foci in two neurodegenerative disorders: *C9orf72* associated ALS and frontotemporal dementia (C9 ALS/FTD) and Fragile X-associated tremor/ataxia syndrome. R-HCR to both G_4_C_2_ and CGG repeats exhibited comparable specificity but > 40 × sensitivity compared to FISH, with better detection of both nuclear and cytoplasmic foci in human C9 ALS/FTD fibroblasts, patient iPSC derived neurons, and patient brain samples. Using R-HCR, we observed that integrated stress response (ISR) activation significantly increased the number of endogenous G_4_C_2_ repeat RNA foci and triggered their selective nuclear accumulation without evidence of stress granule co-localization in patient fibroblasts and patient derived neurons. These data suggest that R-HCR can be a useful tool for tracking the behavior of repeat expansion mRNA in C9 ALS/FTD and other repeat expansion disorders.

**Supplementary Information:**

The online version contains supplementary material available at 10.1186/s40478-021-01169-8.

## Introduction

GC-rich repeat expansions are the genetic cause of over 50 neurodevelopmental, neurodegenerative, and neuromuscular diseases. Repeat expansions elicit disease through multiple mechanisms (*reviewed in* [51, 57]). Repeats as DNA can inhibit transcription of repeat-containing genes and promote the DNA damage response through R-loop formation [25, 27, 54, 55, 73]. Repeats as RNA can functionally sequester both canonical and non-canonical RNA binding proteins and prevent them from performing their normal functions [11, 12, 32, 33, 40, 44, 48, 57, 68]. Repeats can also be translated into toxic proteins, either via canonical translation (as occurs for a number of polyglutamine expansions) or via repeat associated non-AUG (RAN) initiated translation [24, 34, 39, 53, 71, 72, 86].

**Fig. 1:**
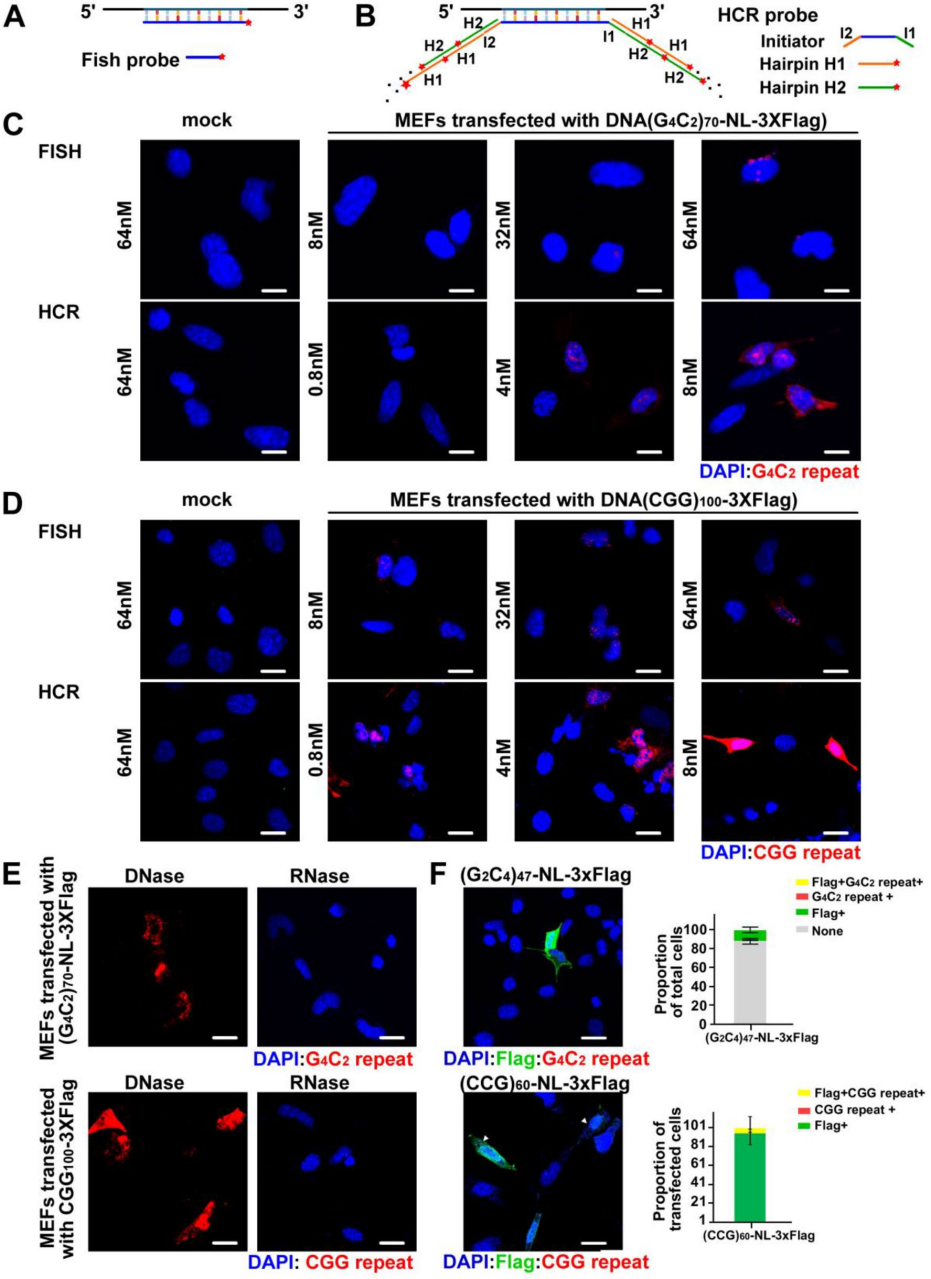
Hybridization chain reaction increases detection of GC rich repeats over FISH. **a** FISH probe with a 5′ conjugated fluorophore. Signal strength is dependent on number of RNA molecules present. **b** In R-HCR, two probe types are used: the initiator probe which binds directly to the RNA of interest, and 5' fluorophore conjugated hairpin probes (H1 and H2) complementary to 3′ and 5′ extensions on the initiator probe. Upon binding, H1 and H2 unfold to reveal new binding sites for the other hairpin probe. In this way, signal from one RNA molecule is amplified > 100 fold, dramatically enhancing detection. **c**, **d** MEFs transfected with (G_4_C_2_)_70_-NL-3xFlag (**c**) or CGG_100_-3xFlag (**d**) vectors and probed with indicated concentrations of either FISH or R-HCR probe. **e** MEFs transfected with (G_4_C_2_)_70_-NL-3xFlag expressing vector (*top*) or CGG_100_-3xFlag vector (*bottom*) and treated with DNase (*left*) or RNase (*right*) prior to R-HCR. **f** ICC-R-HCR of MEFs transfected with antisense (G_2_C_4_)_47_-NL-3xFlag (*top*) or (CCG)_60_-NL-3xFlag (*bottom*) expressing plasmids. *Top*: N = 434; *Bottom*: N = 38.Error bars indicate 95% confidence intervals (CI). Scale bar = 20 μm in **c**, **d**, **e**; 50 μm in **f**

One of the key pathological hallmarks in repeat expansion diseases is the presence of repeat-containing RNA foci. These foci are thought to represent repeat RNAs co-associated with specific RNA binding proteins, although they may also arise from intra- and inter- molecular RNA-RNA interactions via RNA gelation [1, 18, 19, 30–32, 45, 61, 62, 64, 70]. The exact behavior, biophysical properties, and associated protein and RNA factors of these RNA condensates varies across different repeat expansions *(reviewed in* [17, 33, 50]). Traditionally, these foci are visualized by fluorescent in situ hybridization (FISH) (Fig. 1a) [29, 32, 40, 75–77]. Early studies were able to successfully identify repeat containing foci because the repetitive nature of the repeat allowed single probes to “tile” along the mRNA and enhance signal detection. However, FISH in human tissues is sometimes hindered by the low abundance of repeat containing RNAs, and high background due to both auto-fluorescence of tissue and the human transcriptome containing numerous GC rich repeats [2, 14, 15, 37, 60, 67, 75, 81].

Recently, a more sensitive method, hybridization chain reaction (HCR) was developed that uses an initiator probe recognizing the RNA of interest and a pair of hairpin probes conjugated with fluorophores to amplify the initiator signal (Fig. 1b) [6–9]. This approach significantly amplifies the signal of an individual molecule over traditional FISH and makes it easier to detect low abundant RNAs and overcome background signal caused by auto-fluorescence and off-target binding [63]. Here, we utilized HCR to detect RNA foci associated with G_4_C_2_ repeat expansions in *C9orf72* that are the most common genetic cause of ALS and FTD and CGG repeats associated with Fragile X disorders such as Fragile X-associated tremor/ataxia syndrome (FXTAS) [14, 28, 56]. Repeat HCR (R-HCR) provided significantly higher sensitivity for detection of repeats and allowed accurate tracking of endogenous GGGGCC repeat RNAs in patient cells in response to cellular stress activation. Taken together, these data suggest that R-HCR can be a valuable addition to analysis and imaging pipelines in repeat expansion disorders.

## Results

### R-HCR is more sensitive than FISH for detecting GC rich repeats

We first compared the sensitivity and specificity of traditional FISH vs R-HCR probes. We designed fluorophore labeled locked nucleic acid (LNA) (C_4_G_2_)_6_ and (CCG)_8_ FISH probes and (C_4_G_2_)_6_ and (CCG)_10_ R-HCR initiator probes to hybridize to corresponding G_4_C_2_ or CGG repeats. Alexa fluorophore labeled amplifier hairpin probes B1H1 and B1H2 were then used to amplify the R-HCR probe signal (Fig. 1a, b, Additional file 1: Table 2) [7, 9]. We conducted a side-by-side comparison in mouse embryonic fibroblasts (MEFs) expressing (G_4_C_2_)_70_-NL-3xFlag or (CGG)_100_-3xFlag reporters [24, 34]. We found more repeat positive cells by R-HCR than FISH when using the same probe concentration (8 nM) (Fig. 1c, d). When we further increased each FISH probe concentration to 64 nM, we only saw a modest increase in number of repeat positive cells that remained significantly lower than that seen using R-HCR. In contrast, when we decreased each R-HCR probe concentration to 4 nM and 0.8 nM, we still observed enhanced signal compared to FISH, for both G_4_C_2_ and CGG repeats (Fig. 1c, d). Furthermore, both nuclear and cytoplasmic signal was readily detected using R-HCR, while FISH primarily detected only the stronger nuclear signal. No signal was detected by either method in MEFs not expressing G_4_C_2_ or CGG repeats (Fig. 1c, d). Together, these results show that R-HCR is more sensitive than FISH for detecting exogenous GC rich repeats.

To confirm that the observed signal from R-HCR was due to probe hybridization to RNA and not DNA, we treated the (G_4_C_2_)_70_ and (CGG)_100_ transfected MEFs with DNase or RNase prior to R-HCR. While DNase robustly eliminated DAPI signal, it had no effect on the GC-rich repeat signal. Conversely, almost all probe signal went away when cells were treated with RNase (Fig. 1e). To further validate that our R-HCR probes were specifically recognizing their target RNA, we transfected cells with antisense CCCCGG (ATG-(C_4_G_2_)_47_-NL-3xFlag) or CCG ((CCG)_60_-NL-3xFlag) reporters. We did not detect any repeat signal in Flag positive cells (Fig. 1f). Together this supports that our R-HCR probes specifically hybridize to G_4_C_2_ and CGG repeat containing RNA, with high specificity and sensitivity.

**Fig. 2:**
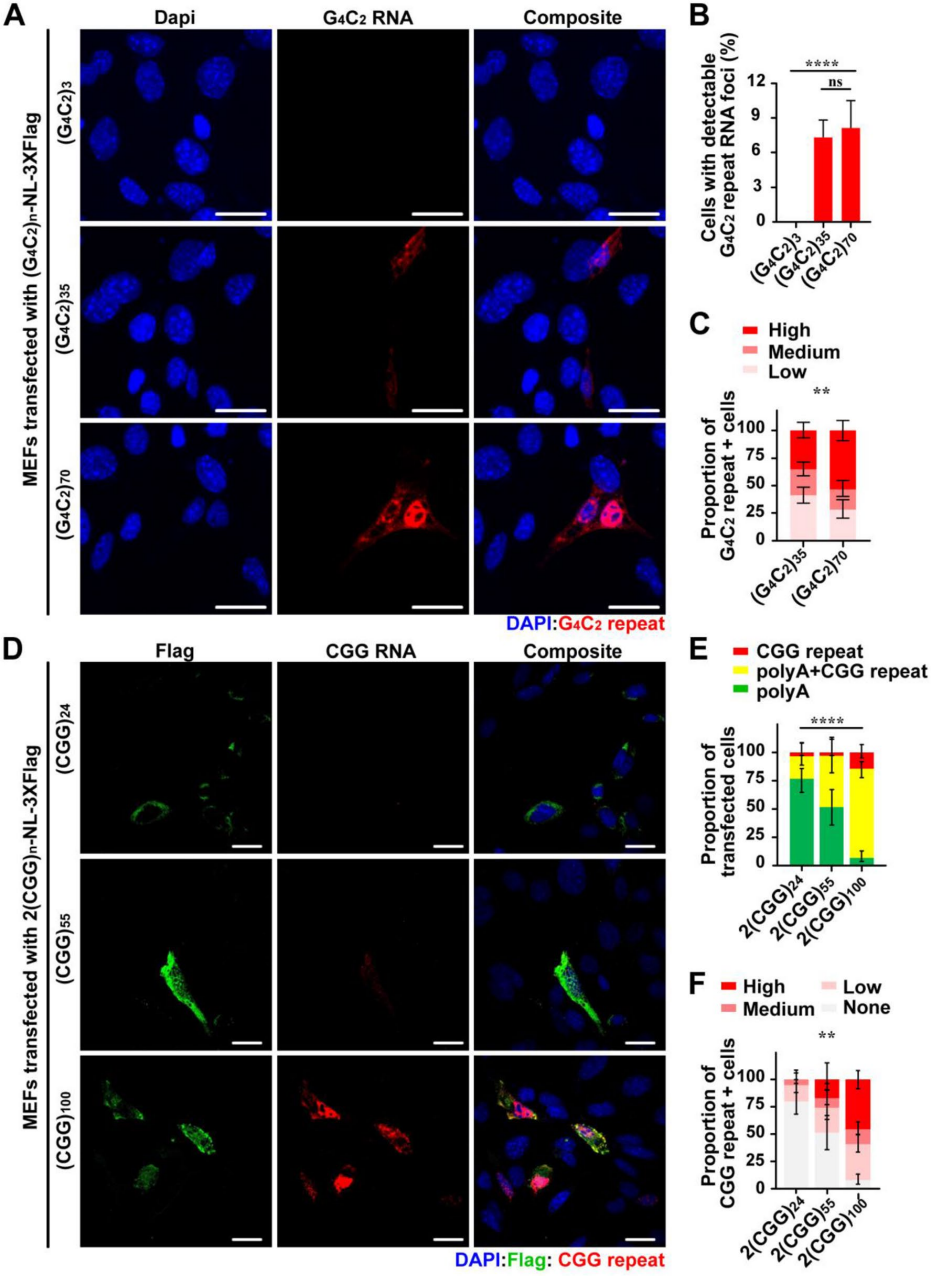
R-HCR probe signal intensity is repeat length dependent for G_4_C_2_ and CGG repeats. **a** R-HCR on MEFs transfected with G_4_C_2_ repeat constructs with increasing repeat sizes. **b** Quantification of cells with detectable G_4_C_2_ repeat RNA foci in total cells. (G_4_C_2_)_3_: N = 313; (G_4_C_2_)_35_ N = 575; (G_4_C_2_)_70_ N = 514. **c** Quantification of R-HCR signal intensity in MEFs transfected with (G_4_C_2_)_n_-NL-3xF reporters with indicated repeat length. (G_4_C_2_)_35_: N = 175; (G_4_C_2_)_70_: N = 110. **d** R-HCR on MEFs transfected with CGG repeat constructs with increasing repeat sizes. **e** Quantification of Flag + and CGG RNA + cells expressed as proportion of total transfected cells. **f** Quantification of R-HCR signal intensity in MEFs transfected with 2(CGG)n-NL-3xF reporters with indicated repeat length. **e-f**N ≥ 35/condition. Error bar indicates 95% CI. Statistics: Chi-square test for **b**, **c**, **e** and **f**. Fisher’s exact test for **b**, **p < 0.01, ****p < 0.0001, ns: not significant. Scale bar = 50 μm in **a** and **d**

We next determined whether R-HCR could distinguish between different G_4_C_2_ repeat sizes (3, 35, or 70 G_4_C_2_ repeats). Despite similar transfection efficiencies among all three conditions (as monitored by GFP co-transfection, Additional file 2: Fig. 1a–c), we observed no signal in cells expressing (G_4_C_2_)_3_-NL-3xFlag. The number of R-HCR positive cells was comparable for both (G_4_C_2_)_35_-NL-3xFlag and (G_4_C_2_)_70_-NL-3xFlag transfection (Fig. 2a, b). However, there was a significant correlation between R-HCR signal intensity and repeat length, with (G_4_C_2_)_70_-NL-3xFlag transfected cells having higher R-HCR signal intensity (Fig. 2c).

We next performed similar experiments in cells transfected with CGG_n_-NL-3xFlag reporters with repeats of various length (24, 55, and 100 CGG repeats)_._ We observed a strong positive correlation between CGG repeat length and the number of R-HCR positive cells (Fig. 2d, e). We also observed a significant increase in R-HCR signal intensity within cells expressing longer CGG repeats (Fig. 2d, f). CGG repeat expansions within the *FMR1* 5′UTR have previously been shown to enhance transcription, and we and others have observed a positive correlation of repeat length and RNA abundance in this setting ([4, 36] and unpublished data). Thus, this repeat-length increase in R-HCR signal could result from enhanced mRNA production as well as more CGG repeat binding sites in the longer repeat reporters. As both repeat size and expression are tightly linked to disease, we believe R-HCR can be used to qualitatively assess CGG repeat RNA burden in model systems.

### R-HCR is more sensitive than FISH at detecting endogenous repeat signal

**Fig. 3:**
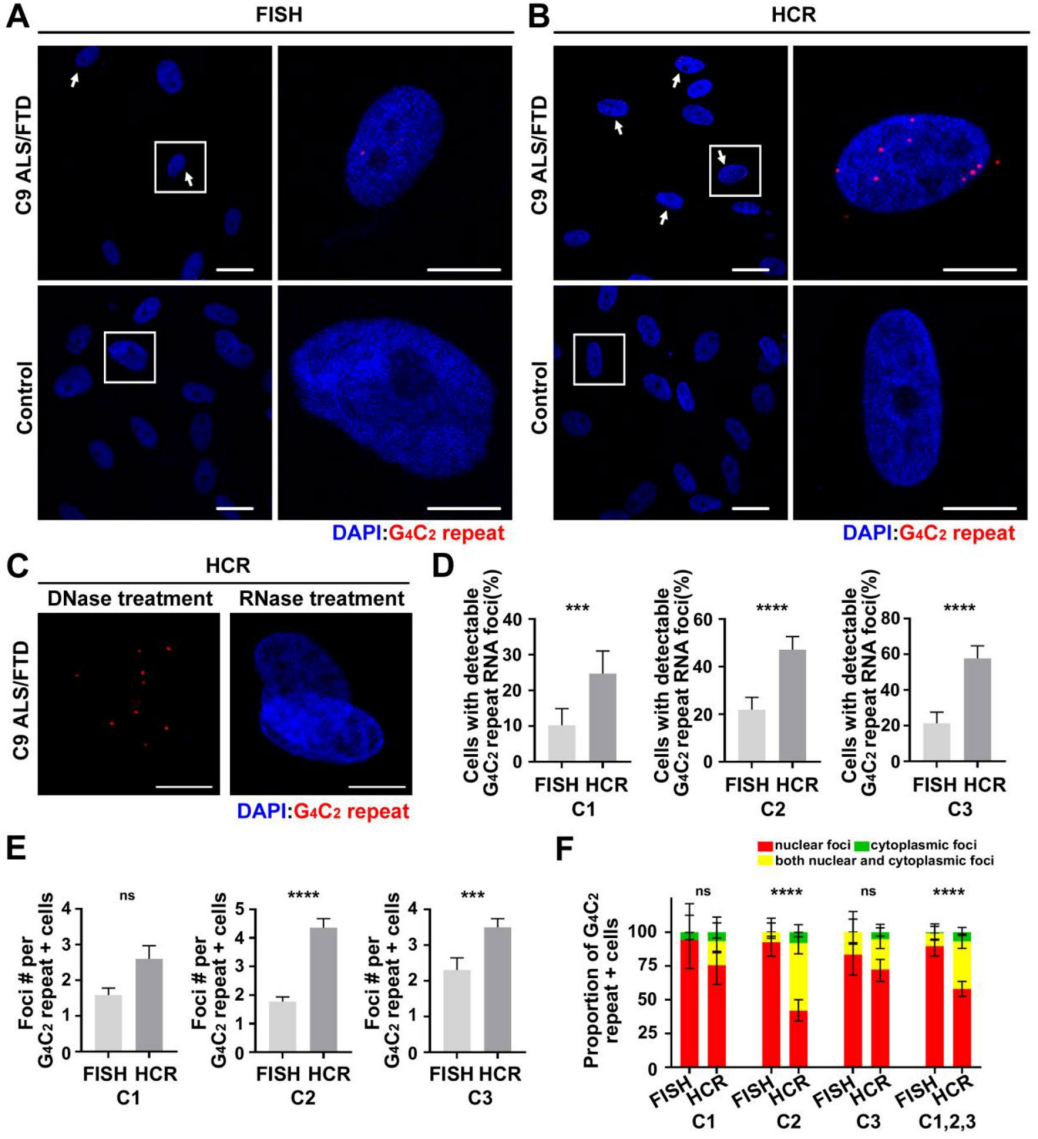
R-HCR is more sensitive than FISH at detecting endogenous G_4_C_2_ repeats in *C9orf72* ALS-FTD patient fibroblasts. **a–c** FISH (**a**) and R-HCR (**b**) in three (C1–C3) *C9orf72* ALS-FTD patient and control fibroblast lines, **c** treated with DNase and RNase prior to R-HCR. White arrows were used indicate RNA foci positive cells in some images. Higher magnification images of the boxed areas are shown on the right. **d** Quantification of **a** and **b**. **e–f** Quantification of RNA foci number per RNA + cell and the distribution for RNA foci. Error bars = 95% CI (**d** and **f** ) and ± SEM (**e**). N > 150 for each line. Statistics: Fisher’s exact test (**d**), unpaired t test (**e**) and Chi-square test (**f**). ***p < 0.001, ****p < 0.0001, ns = not significant. Scale bar = 50 μm in left for **a** and **b**; 20 μm in right for **a** and **b**, and **c**

We next determined if R-HCR was effective at detecting endogenous GC-rich repeat RNA. We first compared R-HCR and FISH in control and *C9orf72* patient fibroblasts. Unlike in transiently transfected cells where RNA signal via R-HCR was predominantly globular and nuclear, endogenous G_4_C_2_ repeats appear primarily as small foci, similar to foci detected by FISH. Previous studies observed upwards of 35% of *C9orf72* patient fibroblasts contained at least 1 G_4_C_2_ repeat foci [42], although signal specificity was not established. Here, after normalizing to control fibroblast signal, we observed only half the number of G_4_C_2_ repeat positive cells previously reported in three different expansion cell lines (C9-C1, C9-C2, C9-C3). However, our number of foci/foci positive cell is in agreement with previously published work (Fig. 3e) [13, 42]. Using R-HCR, we detected > 2 × more G_4_C_2_ positive cells than with FISH (Fig. 3a, b, d). Importantly, we also observed a significant increase in the number of foci/foci positive cell (Fig. 3e). Intriguingly, while FISH primarily detected foci in the nucleus of G_4_C_2_ repeat expansion cell lines, R-HCR was able to detect cytoplasmic foci in ~ 40% of G_4_C_2_ repeat positive cells (Fig. 3f). Previous studies observed cytoplasmic foci only ~ 10% of the time [49]. To confirm that the signal we were observing was from RNA, we treated the G_4_C_2_ repeat expansion fibroblasts with DNase or RNase before R-HCR and found the R-HCR signal was sensitive to RNase and resistant to DNase (Fig. 3c).

**Fig. 4:**
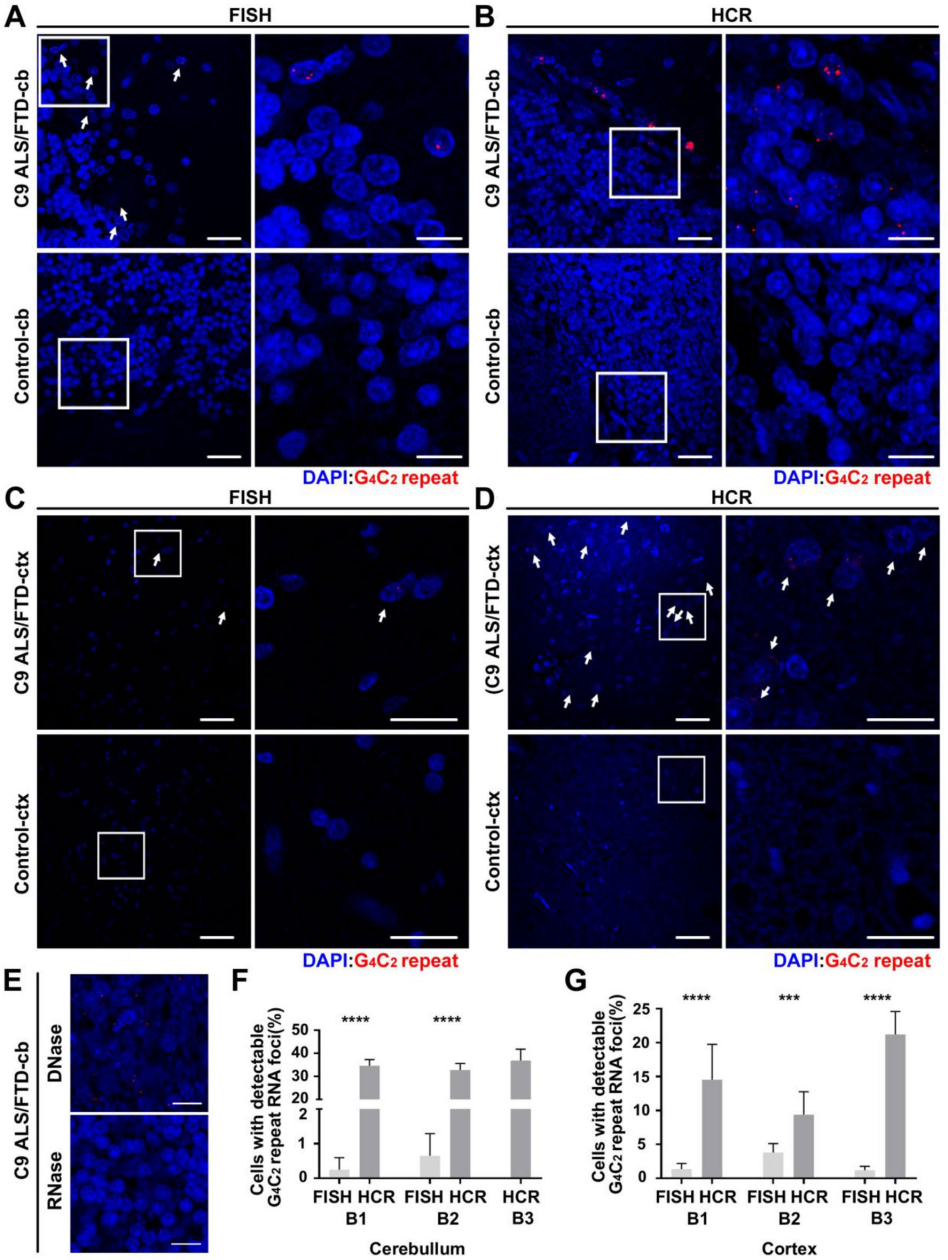
R-HCR is more sensitive than FISH at detecting endogenous G_4_C_2_ repeats in *C9orf72* ALS-FTD patient brains. **a–e** FISH (**a, c**) and R-HCR (**b, d, e**) cerebellum (**a, b, e**) and frontal cortex (**c, d**) from three *C9orf72* ALS-FTD patients and controls. **e** C9 cases treated with DNase and RNase prior to R-HCR. White arrows indicate RNA foci positive cells. Higher magnification images of the boxed areas are shown on the right. **f** Quantification of the percentage of cerebellar granule cells with detectable G_4_C_2_ repeat RNA foci in 3 cases. **g** Quantification of the percentage of cortical neurons and glia with detectable G_4_C_2_ repeat RNA foci in three C9orf72 cases ± 95%CI. N > 150 for each sample. Statistics: Fisher’s exact test for **f** and **g**. ***p < 0.001, ****p < 0.0001. Scale bar = 50 μm in left for **a**–**d**; 20 μm in right for **a**–**e**. cb = cerebellum, ctx = frontal cortex

We next compared FISH and R-HCR in control and *C9orf72* patient brain tissue. We looked for foci in cerebellum and frontal cortex, as those regions have been previously shown to have G_4_C_2_ repeat RNA foci and feature evidence of disease pathology [13, 49]. After normalizing signal to controls, we detected G_4_C_2_ repeat positive cells in the frontal cortex and cerebellum of three C9 brains (C9-B1, C9-B2 and C9-B3) using FISH. However, foci were only detected in less than 1% of granule cells in the cerebellum, and less than 5% of cells (predominantly glia and interneurons) in the frontal cortex. In contrast, when R-HCR was performed on these same brain samples, more than 30% of granule cells were positive for G_4_C_2_ repeat RNA foci in the cerebellum and 8–21% of glia and interneurons were positive for G_4_C_2_ repeat RNA in the frontal cortex (Fig. 4a–d, f–g). These foci were absent when tissue was RNase treated, and remained when tissue was DNase treated, strongly supporting that these are RNA foci (Fig. 4e). R-HCR also enhanced the number of foci/foci + cell in both the cerebellum and frontal cortex, however this difference was only significant in one case (Additional file 2: Fig. 2e, f). We also observed diffuse staining in both purkinje cells and pyramidal neurons with both FISH and R-HCR (Additional file 2: Fig. 2a–d). Together, these data indicate that R-HCR can be useful in detection of low abundant endogenous G_4_C_2_ repeat RNA.

We performed a similar experiment in control and FXTAS patient fibroblasts. With the sense CGG repeat R-HCR probe, we found CGG repeat signal was readily detectable not only in FXTAS patient cells lines, but also in premutation carriers and control lines. The pattern of the detected signal appeared predominantly nucleolar similar to prior reports in FXTAS brain tissue [61, 62, 70]. We did not observe any R-HCR signal in these same cell lines when we used the antisense CCG repeat R-HCR probe or after RNAse treatment, suggesting this signal was primarily CGG RNA-mediated (Additional file 2: Fig. 3a). These results indicate that the CGG repeat signals are CGG RNA-specific but not *FMR1* CGG repeat expansion-specific. We next evaluated R-HCR on CGG repeats in frontal cortex and hippocampal sections as these have been previously shown to express the CGG RAN product, FmrpolyG [38]. Similar to fibroblasts, we observed nucleolar-like staining in both control and FXTAS patient brain tissue with both the CGG FISH and R-HCR probes. However, the signal was stronger and occurred in a higher percentage of neurons in FXTAS samples (Additional file 2: Supplemental Fig. 3b).

As the control cell lines still contain ~ 20–30 CGG repeats, we reasoned that the probe could still be specifically binding to *FMR1* RNA. To investigate this further, we performed R-HCR in a transcriptionally silenced FXS iPSC line with 800 repeats, a control iPSC line with ~ 30 CGG repeats and then compared their signal intensity to an unmethylated full mutation line (TC-43) with a large (270) transcriptionally active CGG repeat expansion that supports RAN translation [26, 58]. We observed significant staining with the CGG R-HCR probe in all three cell lines that was both diffuse in the cytoplasm, as well as localized to the nucleolus (Additional file 2: Fig. 3c). However, similar to observations in FXTAS brain, we saw enhanced signal in the unmethylated full mutation line compared to the WT and methylated FXS line, suggesting that this enhanced signal was due to CGG repeat expansions within *FMR1* (Additional file 2: Fig. 3c–e).

### G4C2 repeats accumulate in the nucleus in response to cellular stress

**Fig. 5:**
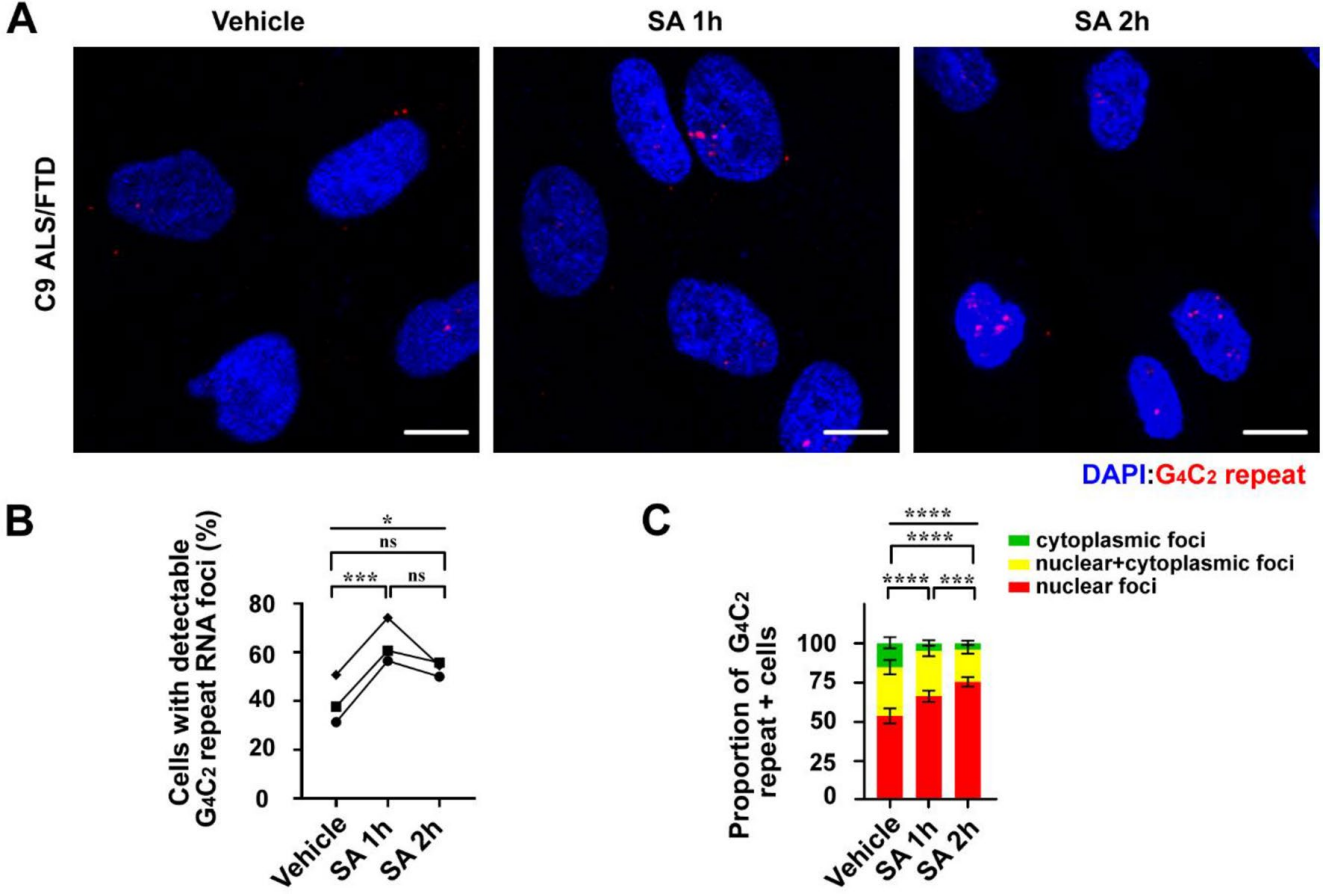
G_4_C_2_ repeat RNAs redistribute in the nucleus during stress in *C9orf72* ALS-FTD patient fibroblasts. **a** R-HCR of *C9orf72* ALS-FTD patient fibroblasts treated with H_2_O (vehicle) or sodium arsenite (SA) for the indicated times. **b)** Quantification of cells with detectable G_4_C_2_ repeat RNA foci in total cells. The dot shapes represent different *C9orf72* patient fibroblast lines. N > 150 for each line for vehicle, SA 1.0 h, and SA 2.0 h. **c** Distribution of G_4_C_2_ repeat RNA foci with or without SA treatment ± 95%CI. N = 400 for vehicle, 679 for SA 1.0 h, and 775 for SA 2.0 h. Statistics: matched one-way ANOVA and paired t test (**b**), and Chi-square test (**c**). *p < 0.05, ***p < 0.001, ****p < 0.0001. *ns* not significant. Scale bar = 10 μm in **a**

**Fig. 6:**
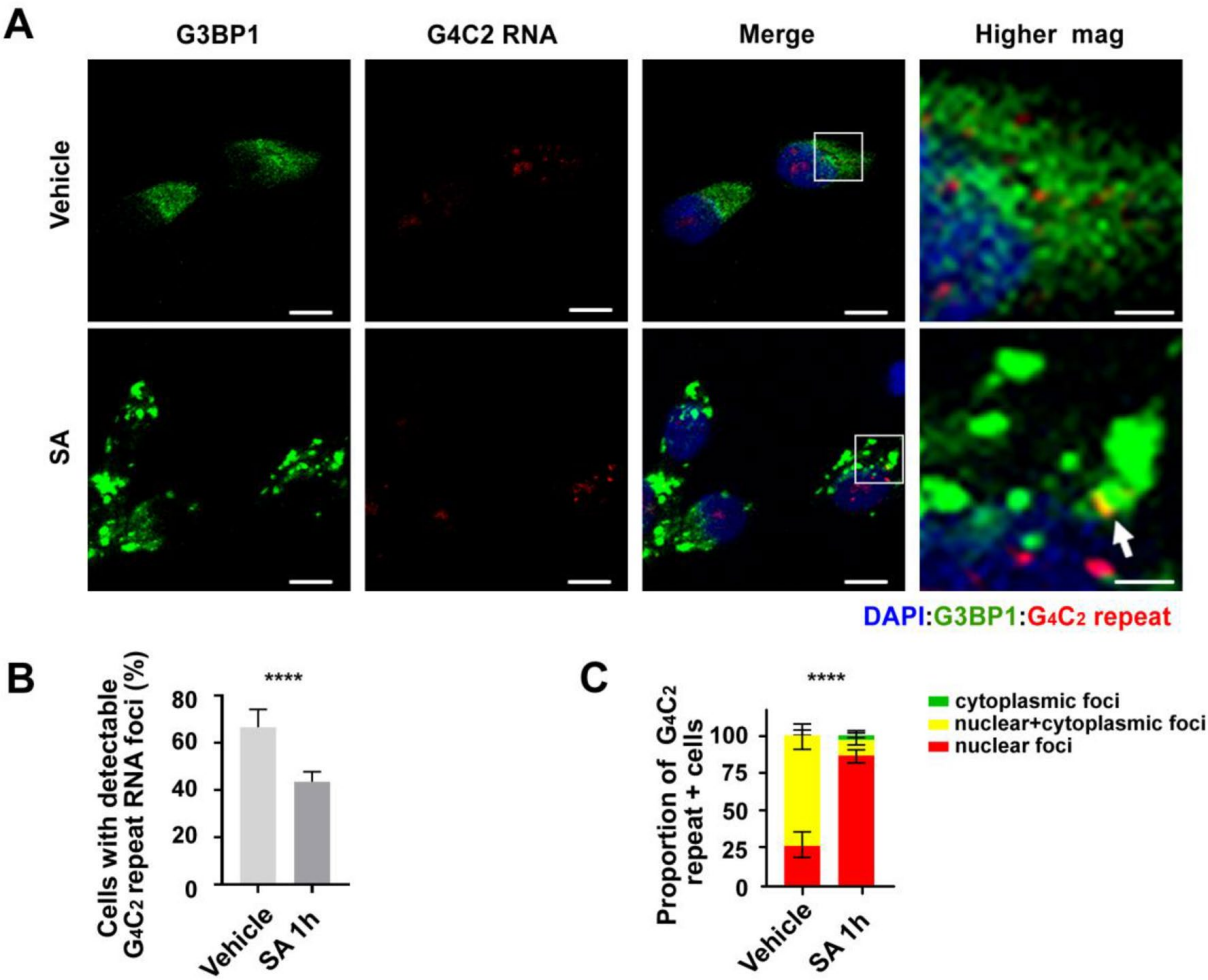
G_4_C_2_ repeat RNAs redistribute in the nucleus during stress in *C9orf72* ALS-FTD patient neurons. **a** R-HCR-ICC of *C9orf72* ALS-FTD patient derived neurons treated with H_2_O or SA. G3BP1 = stress granule marker. A higher magnification of the boxed areas are shown on the right. White arrows indicate rare colocalization events between cytoplasmic G_4_C_2_ repeats and G3BP1. **b** Quantification of (**a**). Bars represent proportion of neurons with detectable G_4_C_2_ repeat RNA among total neurons ± 95%CI. N = 156 for vehicle and 535 for SA 1.0 h. **c** Quantification of the distribution of repeat RNA in all repeat positive neurons ± 95% CI. N = 104 for vehicle and 233 for SA 1.0 h. Statistics: Fisher’s exact test (**b**) and Chi-square test (**c**). ****p < 0.0001. Scale bar = 10 μm in (**a**) except for the zoomed in images (2.5 μm)

The ability to readily detect endogenous nuclear and cytoplasmic G_4_C_2_ repeat RNA foci using R-HCR is potentially useful for exploring its roles in disease pathogenesis. Our lab and others have observed that expression of G_4_C_2_ repeat containing reporters induces stress granule (SG) formation and the integrated stress response (ISR), and considerable evidence now suggests that this process can contribute to neurodegeneration [24, 41, 65, 84]. Moreover, exogenous ISR activation through a variety of methods triggers a selective enhancement of RAN translation from both CGG and G_4_C_2_ repeats in transfected cells and neurons [5, 24, 65, 80]. To investigate the behavior of endogenous G_4_C_2_ repeat RNA and foci in response to stress, we treated *C9orf72* patient fibroblasts with sodium arsenite (SA) or vehicle. SA treatment for one or two hours led to a significant increase in the total number of cells with visible G_4_C_2_ repeat foci. Moreover, there was a marked re-distribution of these foci into the nucleus and out of the cytoplasm (Fig. 5a–c). This same SA-induced nuclear re-distribution of G_4_C_2_ repeat foci was also observed in *C9orf72* patient derived neurons (Fig. 6a–c).

**Fig. 7:**
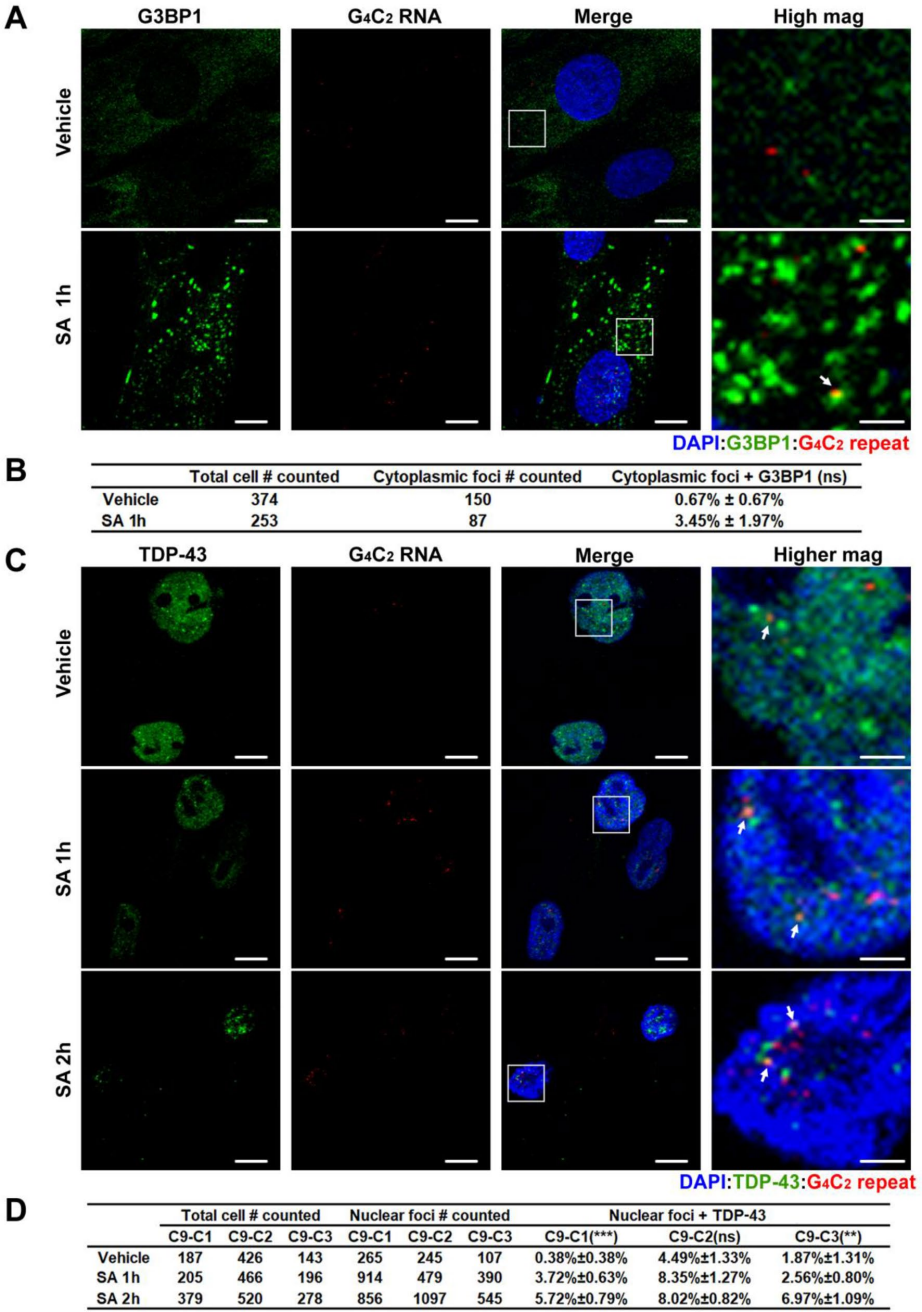
G_4_C_2_ repeat RNAs rarely co-localize with cytoplasmic G3BP1 or nuclear TDP-43 during stress. **a** R-HCR-ICC of *C9orf72* patient derived fibroblasts treated 1 h with H_2_O or SA. G3BP1 = stress granule marker. Higher magnification images of the boxed areas are shown on the right. White arrows indicate co-localization of G_4_C_2_ repeat and G3BP1. **b** Quantification of cytoplasmic G_4_C_2_ repeat foci co-localization with G3BP1 as a fraction of all cytoplasmic G_4_C_2_ repeats. **c** R-HCR-ICC of *C9orf72* patient derived fibroblasts treated with H_2_O or SA (1 h, 2 h). TDP-43 = nuclear body marker. White arrows indicate co-localization of G_4_C_2_ repeat and TDP-43. **d** Quantification of nuclear G_4_C_2_ repeat foci colocalization with TDP-43 as a fraction of all observed nuclear G_4_C_2_ repeat foci. % shown as proportion of co-localization for each condition ± SEM. Statistics: Fisher’s exact test (**b**) and Chi-square test (**d**). **p < 0.01, ***p < 0.001. ns: not significant. Scale bar = 10 μm in (**a**) and (**c**) except for zoomed images (2.5 μm)

RNAs typically move into SGs and become translationally silenced in response to SA stress [35]. In contrast, G_4_C_2_ repeat RNAs remain translationally competent after stress induction. We therefore assayed whether G_4_C_2_ repeat RNA foci localized to SGs in response to stress. Consistent with their retained translational competency, we did not observe significant co-localization of G_4_C_2_ repeat foci with the SG marker, G3BP1 (Fig. 7a, b), although rare co-localization events were observed.

Integrated stress response activation induces the formation of nuclear stress bodies. These TDP-43 positive structures are thought to contribute to ALS disease pathogenesis [78]. Given the greater nuclear distribution of G_4_C_2_ repeat RNA after stress induction, we evaluated whether there was any significant overlap between endogenous G_4_C_2_ repeat RNA and TDP-43, a critical factor in *C9orf72* ALS/FTD pathology and ALS pathogenesis as well as a robust marker for nuclear bodies [16, 78]. Our untreated C9 fibroblasts appeared to have small TDP-43 nuclear bodies, indicative of them being inherently stressed. Upon 2 h of SA induction we saw a decrease in diffuse nuclear TDP-43, and an increase in TDP-43 nuclear foci size. Overall, nuclear TDP-43 showed limited (0.38–4.49%) co-localization with G_4_C_2_ repeat RNA foci at baseline. After SA induction, there was a significant increase in this co-localization, but it remained modest (2.56% ~ 8.35%) (Fig. 7c, d). Taken together, these studies suggest that nuclear retention or re-distribution of G_4_C_2_ repeat RNA foci in *C9orf72* fibroblasts in response to stress is not predominantly driven by either SG or nuclear body association and may instead reflect nucleocytoplasmic transport defects elicited by stress pathway activation [3, 43, 52, 84].

## Discussion

Repeat RNA and the formation of RNA–protein and RNA-RNA condensates are thought to act as significant factors in the pathogenesis of multiple repeat expansion disorders. However, traditional detection techniques such as FISH are often limited in their sensitivity which may cloud the roles of such repeat RNAs in disease-relevant processes. Here we used a highly sensitive RNA in situ amplification method, R-HCR, to readily detect low expressing endogenous GC-rich repeat expansions in both patient cells and tissues. This non-proprietary method provided significantly enhanced sensitivity over RNA FISH probes with retained specificity. This increased sensitivity allowed for greater detection and appreciation of nuclear and cytoplasmic foci in patient cells. Moreover, we demonstrated that G_4_C_2_ repeat RNA foci accumulate in the nucleus in both patient fibroblasts and neurons in response to cellular stress. This tool should prove useful to the field in explorations of endogenous repeat RNA behaviors and pathology in both repeat expansion disorders and model systems.

Previously established techniques for detecting RNA in situ have limitations. FISH is limited by RNA copy number, and probe specificity, while the use of MS2 and PP7 binding sites to detect low abundant RNAs is only applicable to exogenous gene expression, or in cases where these tags were inserted via CRISPR [20, 66, 69, 82]. Recently, an alternative RNA in situ amplification method, BaseScope™, was shown to improve detection of endogenous G_4_C_2_ repeats in patient tissue [46]. R-HCR and BaseScope™ are comparable on a number of fronts. Namely, they both can be combined with IHC, have extensive signal amplification capacity, and in the case of newer R-HCR versions, utilize split probes to eliminate nonspecific signal [8]. However, R-HCR lends itself as a more universally applicable approach for a number of reasons, including minimal optimization needed, fewer steps involved, flexibility in hybridization temperature (and thus probe stringency), and the option of five different fluorophores to allow for combined R-HCR-IF with multiple probes and/or antibodies. However, while the flexibility of fluorescent probe choice makes R-HCR more adaptable in a variety of experimental settings, the chromogenic properties of BaseScope™ may allow for better coupling with tissue stains for pathology purposes. Thus, both serve as valuable tools for detecting and investigating endogenous GC-rich repeats.

While both R-HCR and BaseScope™ are sensitive tools for detecting G_4_C_2_ repeat RNA, we do caution the use of these techniques for detecting CGG repeat RNA. Given the extensive use of CGG RNA FISH probes in the literature, we were surprised to find such high background signal, specifically in control and FXS human samples. The staining pattern with the CGG probe was also vastly different from the foci typically observed for other GC-rich repeat expansions. In fibroblasts, brain samples, and HEK293 cells we observed intense, large nuclear body staining, while iPSCs had weaker nuclear body staining and strong, diffuse cytoplasmic staining. This pattern is largely consistent with prior work using FISH to assay CGG repeat RNA in FXTAS [61, 62]. That lack of punctate RNA foci makes it difficult to determine what signal is specific to the CGG repeat expansion on *FMR1*. There are 921 human genes which contain ≥ 6 CGG repeats, and this likely accounts for the high background in human cells [26, 37]. However, previous studies using probes to the 3′ UTR and coding sequence of *FMR1* showed similar large nuclear body staining, suggesting the signal observed with our CGG R-HCR probe could still be disease relevant [70].

Our R-HCR G_4_C_2_ probe exhibited significantly better sensitivity and specificity in human cells and tissues. The increased sensitivity of R-HCR over FISH with this probe allowed us to consistently visualize G_4_C_2_ repeat RNA in the cytoplasm, allowing us to ask questions regarding G_4_C_2_ repeat RNA activities in different subcellular compartments. As a proof of concept, we analyzed G_4_C_2_ repeat cellular localization during stress. We observed no significant co-localization with SGs, but instead, a redistribution of G_4_C_2_ foci into the nucleus. This redistribution could either be caused by an increase in nuclear import, a decrease in nuclear export, or a retention of repeat RNA within sub-nuclear compartments. Nucleocytoplasmic transport is inhibited basally in many neurodegenerative conditions, including *C9orf72* ALS/FTD [59]. Nucleocytoplasmic transport proteins, including importin-alpha, RanGap, and nucleoporins, are also recruited into SGs and co-localize with TDP-43 in ALS/FTD mutant cytoplasmic aggregates [10, 23]. SG assembly itself inhibits nucleocytoplasmic transport by sequestering factors required for nuclear export, and thus the increased abundance of G_4_C_2_ repeat RNA may be indicative of global nuclear mRNA retention [3, 43, 52, 84]. G_4_C_2_ repeat RNA itself is also implicated in nuclear import perturbations via binding to RanGap1 [85], suggesting that its nuclear retention could be not only a cause of but also a contributor to stress-dependent pathology.

Alternatively, repeat RNAs may interact with nuclear stress bodies. These complexes result from nuclear re-localization of heat shock factors, including HSF1 and HSP70, as well as RNA factors, including TDP-43, with satellite III repeat RNAs [22, 47, 74, 83]. We observed a significant increase in co-localization between nuclear TDP-43 and G_4_C_2_ RNA. However, the overall overlap between these two molecules remained modest and of unclear biological significance.

In sum, we describe the application of R-HCR to the detection of endogenous GC rich repeat RNA. This non-proprietary tool is sensitive, specific and useful in studying endogenous repeat RNA foci dynamics and should prove useful for investigators interested in the behavior of these disease-associated RNA species.

## Methods

### Cell lines culture and Clinical Specimens

MEFs were received from Randal Kaufman (Sanford Burnham Prebys Medical Discovery Institute) and cultured in RPMI1640 with 10% fetal bovine serum (FBS) and 1% penicillin/streptomycin (P/S). FXTAS skin fibroblasts were from Paul Hagerman (UC Davis) and University of Michigan donors. *C9orf72* ALS/FTD skin fibroblasts were from Eva Feldman (University of Michigan). Human derived skin fibroblasts were cultured in high glucose DMEM with 10% FBS, 1% non-essential amino acid (NEAA) and 1% P/S. All cells were cultured at 37 °C. Control and FXTAS human brain paraffin sections were obtained from the University of Michigan Brain Bank and are previously described [38, 71]. Human derived neurons were generated and differentiated as previously described [21, 79]. Details regarding FXS Human derived iPSCs have been previously published [26]. Details on these specimens were presented in Additional file 1: Table 1.

### Reporters

The RAN translation reporters 2(CGG)_n_-NL-3xF, (G_4_C_2_)_n_-NL-3xFlag, and (CCG)_60_-NL-3xF were previously published [24, 34, 38]. The (G_2_C_4_)_47_-NL-3xFlag reporter was made by cloning the reporter sequence between NheI and PspOMI in pcDNA3.1 + (Additional file 1: Table 3). The FMRpolyG_100_- RAN translation reporters was made by cloning in the reporter sequence into pcDNA3.1 + between BamHI and PspOMI (Additional file 1: Table 3).

### Transfection

MEFs were transfected according to manufacturer’s protocol (114–15, Polyplus transfection). In brief, MEFs were suspended and incubated with 3:1 Jetprime to 0.25 µg plasmid DNA at 37 °C for 20 min, then seeded onto chamber slides. Cells were fixed 24 h later for ICC-R-HCR.

### Stress treatment

Fibroblasts and neurons were treated with 500 nM sodium arsenite (SA) or vehicle (H_2_O) at indicated time points then washed 1 × in 1xPBS and fixed for ICC-R-HCR.

### ICC

ICC was performed after fixing cells and prior to performing R-HCR, using a modified protocol to one previously described. In brief, following a fix in 4% PFA, cells were further fixed O/N in 70% ethanol, and rehydrated with 1xPBS for 1 h, prior to addition of antibodies. Following the final washes after the secondary, cells were fixed again in 4% PFA for 10 min to fix the secondary antibodies in place. The following antibodies were used: Flag M2 (1:100, F1804, Sigma), GFP (1:500, ab6556, abcam), G3BP1 (1:200, BDB611127, BD Bioscience/Fisher), TDP-43(1:100, 10,782–2-AP, Protein Tech), Alexa Fluor 488 goat anti-mouse IgG (1:500, A11029, Invitrogen) and anti-rabbit IgG (1:500, A11008, Invitrogen).

### FISH

Fluorophore TYE665 labeled locked nuclear acid (LNA) (C_4_G_2_)_6_ and (CCG)_8_ probes (Additional file 1: table 2) were synthesized by Qiagen. The FISH protocol was adapted from [13] and used R-HCR buffers [7, 9]. In brief, MEFs and fibroblasts, were washed with 1 × PBS-MC, fixed in 4% PFA for 10 min, washed 3 times with 1 × PBS and permeabilized in 0.1% TritonX-100 for 15 min. Cells were washed with 1 × PBS, then dehydrated with 70% ethanol for 1 min, 95% for 2 min, then 100% twice for 2 min. Cells were air-dried, then preheated at corresponding hybridization temperature (see below) in probe hybridization buffer (50% formamide, 5 × sodium chloride sodium citrate (SSC), 9 mM citric acid (pH 6.0), 0.1% Tween 20, 50 μg/mL heparin, 1 × Denhardt’s solution, 10% dextran sulfate) for 30 min. The FISH probes were denatured at 80 °C for 2 min before immediately snap cooling in cold hybridization buffer to prevent intermolecular annealing. Cells were incubated in hybridization buffer with 8 nM, 32 nM or 64 nM probes at 71 °C for (C_4_G_2_)_6_ and 66 °C for (CCG)_8_ for 12-16hrs, washed 4 times for 5 min in 5xSSCT (5 × SSC, 0.1% Tween 20) at room temperature (RT), then mounted with ProLong Gold antifade mountant with DAPI.

For human brain paraffin sections, slides were first deparaffinized with xylenes and then rehydrated from 100% ethanol, 95% ethanol, 70% ethanol, 50% ethanol to DEPC treated H_2_O. Rehydrated slides were incubated in 0.3% Sudan black for 5 min followed by proteinase K treatment at RT for 10 min. Permeabilization, preheating, hybridization with probes, washing and mounting are the same as above.

### R-HCR

The initiator probes (CCCCGG)_6_ and (CCG)_10_, were synthesized by OligoIDT. The fluorophore 647 labeled hairpin probes (B1H1 and B1H2) [7] were synthesized by Molecular Instruments. For transfected MEFs and fibroblasts, cells were processed according to Molecular Instrument’s protocol. In brief, cells were fixed in 70% cold ethanol overnight at 4 °C. When R-HCR was performed following ICC, this step occurred prior to ICC. Cells were then preheat in hybridization buffer at 45 °C for 30 min, and incubated with 0.8 nM, 4 nM or 8 nM initiator probe ((CCCCGG)_6_ or (CCG)_10_) at 45 °C incubator overnight. Cells were washed 4 times for 5 min each with pre-warmed probe wash buffer (50% formamide, 5 × SSC, 9 mM citric acid (pH 6.0), 0.1% Tween 20, 50 μg/mL heparin) at 45 °C and 2 × for 5 min with 5 × SSCT at RT. Cells were incubated with snap cooled hairpins B1H1 and B1H2 at room temperature for 12–16 h in amplification buffer. The concentrations of each hairpin (0.375 pmol, 1.875 pmol, and 3.75 pmol per well in an 8 well chamber slide) was proportional to the amount of initiator probe used (0.8 nM, 4 nM or 8 nM). Cells were washed 5 × for 5 min at RT with 5 × SSCT, then mounted with ProLong Gold antifade mountant with DAPI.

For human derived brain section, samples were processed according to Molecular Instrument’s protocol. In brief, Histo-Clear II was used to deparaffinize tissue, then samples were rehydrated and treated with proteinase K as described for FISH. Slides were washed in 1 × TBST, incubated in 0.2 N HCL for 20 min at RT, washed 5 × in 5xSSCT, then incubated in 0.1 M triethanolamine-R-HCR (pH 8.0) with acetic anhydride for 10 min, and washed in 5 × SSCT for 5 min. Slides were preheated and incubated in hybridization buffer with probes at 45 °C in humidity chambers for 12–16 h. The remaining steps were the same as above for R-HCR in cell culture.

(7) DNase and RNase treatments.

Cells (transfected MEFs and fibroblasts) and human brain tissue were treated with Turbo DNase (0.08u/μl) and PureLink RNase A (100 ng/μl) at 37 °C for 30 min following permeabilization, and rehydration steps respectively.

### Imaging and analysis

Images were taken on an Olympus FV1000 confocal microscope equipped with a 40 × oil objective (60 × for iPSC images) and analyzed using ImageJ software. Signal for protein and repeat RNA were normalized to non-transfected cells or control samples.

For brain tissue, we analyzed layer 4–6 prefrontal cortex gray matter and cerebellar lobules 4–5, with focus on the granule cell layer and purkinje cell layer than the molecular layer. For the cerebellum, most of the foci were found in granule cells and thus we limited our analysis to granule cells in this region. We observed additional diffuse staining with some foci detected within purkinje cells as well as foci present at lower frequency in basket cells within the molecular layer. For one case (B3) there was not sufficient tissue to complete analysis with both FISH and HCR, so this was quantified but not used in statistical analyses. For the cortex, there was staining in both neurons and glia, with foci more abundant by both HCR and FISH in glia and inter-neurons. Detectable signal was also present in pyramidal neurons, but for FISH in particular it was difficult to discern foci in these cells compared to a more diffuse signal in the nuclei and peri-nuclear regions. We therefore focused our comparative analysis to foci within the smaller interneuron and glial nuclei and cytoplasm.

Total cell number, protein stained cell number, RNA positive cell number, and foci number and distribution per cell were all manually counted. Signal intensities for iPSC images (Additional file 2: Fig. 3e) were calculated as mean intensity/area using ImageJ. For transfected MEFs, RNA intensity was graded into high, medium and low signal intensity. The ratio of repeat positive cells was calculated as number of RNA positive cells to total cells. The ratio of foci number per cell was calculated as foci number in all repeat positive cells divided by all repeat positive cells. The repeat distribution was expressed as proportion of cells with foci (only nuclear, only cytoplasmic and both) among all repeat positive cells. The relationship between repeat foci and SGs was analyzed as the proportion of co-localization of cytoplasmic G_4_C_2_ repeat foci with G3BP1 granule to total cytoplasmic G_4_C_2_ repeat foci. Similarly, the relationship between repeat foci and NBs was analyzed as the proportion of nuclear G_4_C_2_ repeat foci co-localizing with TDP-43 granules.

### Statistical analysis

All statistical analyses were performed in GraphPad Prism software. Chi-square test was applied for categorical data, including amount of protein and repeat RNA expression in cells in transfected MEFs, repeat RNA signal intensity in transfected MEFs, and distribution of repeat RNA foci in cells. Paired t-test, unpaired t-test and one-way ANOVA were performed to analyze continuous data, including number of detectable repeat foci, foci number per cell, and the co-association rates between repeat RNA and SG or NB markers. We designated P < 0.05 as our threshold for significance with corrections for multiple comparisons.

## Supplementary Information

**Additional file 1**. Supplemental Figures.

**Additional file 2**. Supplemental Tables.

## References

[1] Bajc Cesnik A, Darovic S, Prpar Mihevc S, Stalekar M, Malnar M, Motaln H, Lee YB, Mazej J, Pohleven J, Grosch M, Modic M, Fonovic M, Turk B, Drukker M, Shaw CE, Rogelj B (2019) Nuclear RNA foci from C9ORF72 expansion mutation form paraspeckle-like bodies. J Cell Sci 132. 10.1242/jcs.224303 30745340

[2] Belzil VV, Bauer PO, Prudencio M, Gendron TF, Stetler CT, Yan IK, Pregent L, Daughrity L, Baker MC, Rademakers R, Boylan K, Patel TC, Dickson DW, Petrucelli L (2013). Reduced C9orf72 gene expression in c9FTD/ALS is caused by histone trimethylation, an epigenetic event detectable in blood. Acta Neuropathol.

[3] Burgess HM, Richardson WA, Anderson RC, Salaun C, Graham SV, Gray NK (2011). Nuclear relocalisation of cytoplasmic poly(A)-binding proteins PABP1 and PABP4 in response to UV irradiation reveals mRNA-dependent export of metazoan PABPs. J Cell Sci.

[4] Chen LS, Tassone F, Sahota P, Hagerman PJ (2003). The (CGG)n repeat element within the 5' untranslated region of the FMR1 message provides both positive and negative cis effects on in vivo translation of a downstream reporter. Hum Mol Genet.

[5] Cheng W, Wang S, Mestre AA, Fu C, Makarem A, Xian F, Hayes LR, Lopez-Gonzalez R, Drenner K, Jiang J, Cleveland DW, Sun S (2018). C9ORF72 GGGGCC repeat-associated non-AUG translation is upregulated by stress through eIF2alpha phosphorylation. Nat Commun.

[6] Choi HM, Beck VA, Pierce NA (2014). Multiplexed in situ hybridization using hybridization chain reaction. Zebrafish.

[7] Choi HM, Beck VA, Pierce NA (2014). Next-generation in situ hybridization chain reaction: higher gain, lower cost, greater durability. ACS Nano.

[8] Choi HMT, Schwarzkopf M, Fornace ME, Acharya A, Artavanis G, Stegmaier J, Cunha A, Pierce NA (2018) Third-generation in situ hybridization chain reaction: multiplexed, quantitative, sensitive, versatile, robust. Development 145 10.1242/dev.165753 PMC6031405 29945988

[9] Choi J, Love KR, Gong Y, Gierahn TM, Love JC (2011). Immuno-hybridization chain reaction for enhancing detection of individual cytokine-secreting human peripheral mononuclear cells. Anal Chem.

[10] Chou CC, Zhang Y, Umoh ME, Vaughan SW, Lorenzini I, Liu F, Sayegh M, Donlin-Asp PG, Chen YH, Duong DM, Seyfried NT, Powers MA, Kukar T, Hales CM, Gearing M, Cairns NJ, Boylan KB, Dickson DW, Rademakers R, Zhang YJ, Petrucelli L, Sattler R, Zarnescu DC, Glass JD, Rossoll W (2018). TDP-43 pathology disrupts nuclear pore complexes and nucleocytoplasmic transport in ALS/FTD. Nat Neurosci.

[11] Conlon EG, Lu L, Sharma A, Yamazaki T, Tang T, Shneider NA, Manley JL (2016) The C9ORF72 GGGGCC expansion forms RNA G-quadruplex inclusions and sequesters hnRNP H to disrupt splicing in ALS brains. Elife 5 10.7554/eLife.17820 PMC5050020 27623008

[12] Cooper-Knock J, Walsh MJ, Higginbottom A, Robin Highley J, Dickman MJ, Edbauer D, Ince PG, Wharton SB, Wilson SA, Kirby J, Hautbergue GM, Shaw PJ (2014). Sequestration of multiple RNA recognition motif-containing proteins by C9orf72 repeat expansions. Brain.

[13] DeJesus-Hernandez M, Finch NA, Wang X, Gendron TF, Bieniek KF, Heckman MG, Vasilevich A, Murray ME, Rousseau L, Weesner R, Lucido A, Parsons M, Chew J, Josephs KA, Parisi JE, Knopman DS, Petersen RC, Boeve BF, Graff-Radford NR, de Boer J, Asmann YW, Petrucelli L, Boylan KB, Dickson DW, van Blitterswijk M, Rademakers R (2017). In-depth clinico-pathological examination of RNA foci in a large cohort of C9ORF72 expansion carriers. Acta Neuropathol.

[14] DeJesus-Hernandez M, Mackenzie IR, Boeve BF, Boxer AL, Baker M, Rutherford NJ, Nicholson AM, Finch NA, Flynn H, Adamson J, Kouri N, Wojtas A, Sengdy P, Hsiung GY, Karydas A, Seeley WW, Josephs KA, Coppola G, Geschwind DH, Wszolek ZK, Feldman H, Knopman DS, Petersen RC, Miller BL, Dickson DW, Boylan KB, Graff-Radford NR, Rademakers R (2011). Expanded GGGGCC hexanucleotide repeat in noncoding region of C9ORF72 causes chromosome 9p-linked FTD and ALS. Neuron.

[15] Didiot MC, Ferguson CM, Ly S, Coles AH, Smith AO, Bicknell AA, Hall LM, Sapp E, Echeverria D, Pai AA, DiFiglia M, Moore MJ, Hayward LJ, Aronin N, Khvorova A (2018). Nuclear Localization of Huntingtin mRNA Is Specific to Cells of Neuronal Origin. Cell Rep.

[16] Duan Y, Du A, Gu J, Duan G, Wang C, Gui X, Ma Z, Qian B, Deng X, Zhang K, Sun L, Tian K, Zhang Y, Jiang H, Liu C, Fang Y (2019). PARylation regulates stress granule dynamics, phase separation, and neurotoxicity of disease-related RNA-binding proteins. Cell Res.

[17] Echeverria GV, Cooper TA (2012). RNA-binding proteins in microsatellite expansion disorders: mediators of RNA toxicity. Brain Res.

[18] Fardaei M, Rogers MT, Thorpe HM, Larkin K, Hamshere MG, Harper PS, Brook JD (2002). Three proteins, MBNL, MBLL and MBXL, co-localize in vivo with nuclear foci of expanded-repeat transcripts in DM1 and DM2 cells. Hum Mol Genet.

[19] Fay MM, Anderson PJ, Ivanov P (2017). ALS/FTD-Associated C9ORF72 Repeat RNA Promotes Phase Transitions In Vitro and in Cells. Cell Rep.

[20] Ferguson ML, Larson DR (2013). Measuring transcription dynamics in living cells using fluctuation analysis. Methods Mol Biol.

[21] Fernandopulle MS, Prestil R, Grunseich C, Wang C, Gan L, Ward ME (2018). Transcription factor-mediated differentiation of human iPSCs into neurons. Curr Protoc Cell Biol.

[22] Frottin F, Schueder F, Tiwary S, Gupta R, Korner R, Schlichthaerle T, Cox J, Jungmann R, Hartl FU, Hipp MS (2019). The nucleolus functions as a phase-separated protein quality control compartment. Science.

[23] Gasset-Rosa F, Lu S, Yu H, Chen C, Melamed Z, Guo L, Shorter J, Da Cruz S, Cleveland DW (2019). Cytoplasmic TDP-43 de-mixing independent of stress granules drives inhibition of nuclear import, loss of nuclear TDP-43, and cell death. Neuron.

[24] Green KM, Glineburg MR, Kearse MG, Flores BN, Linsalata AE, Fedak SJ, Goldstrohm AC, Barmada SJ, Todd PK (2017). RAN translation at C9orf72-associated repeat expansions is selectively enhanced by the integrated stress response. Nat Commun.

[25] Groh M, Lufino MM, Wade-Martins R, Gromak N (2014). R-loops associated with triplet repeat expansions promote gene silencing in Friedreich ataxia and fragile X syndrome. PLoS Genet.

[26] Haenfler JM, Skariah G, Rodriguez CM, Monteiro da Rocha A, Parent JM, Smith GD, Todd PK (2018). Targeted reactivation of FMR1 Transcription in fragile x syndrome embryonic stem cells. Front Mol Neurosci.

[27] Haeusler AR, Donnelly CJ, Periz G, Simko EA, Shaw PG, Kim MS, Maragakis NJ, Troncoso JC, Pandey A, Sattler R, Rothstein JD, Wang J (2014). C9orf72 nucleotide repeat structures initiate molecular cascades of disease. Nature.

[28] Hagerman RJ, Leehey M, Heinrichs W, Tassone F, Wilson R, Hills J, Grigsby J, Gage B, Hagerman PJ (2001). Intention tremor, parkinsonism, and generalized brain atrophy in male carriers of fragile X. Neurology.

[29] Hoem G, Raske CR, Garcia-Arocena D, Tassone F, Sanchez E, Ludwig AL, Iwahashi CK, Kumar M, Yang JE, Hagerman PJ (2011). CGG-repeat length threshold for FMR1 RNA pathogenesis in a cellular model for FXTAS. Hum Mol Genet.

[30] Iwahashi CK, Yasui DH, An HJ, Greco CM, Tassone F, Nannen K, Babineau B, Lebrilla CB, Hagerman RJ, Hagerman PJ (2006). Protein composition of the intranuclear inclusions of FXTAS. Brain.

[31] Jain A, Vale RD (2017). RNA phase transitions in repeat expansion disorders. Nature.

[32] Jin P, Duan R, Qurashi A, Qin Y, Tian D, Rosser TC, Liu H, Feng Y, Warren ST (2007). Pur alpha binds to rCGG repeats and modulates repeat-mediated neurodegeneration in a Drosophila model of fragile X tremor/ataxia syndrome. Neuron.

[33] Johnson SJ, Cooper TA (2021). Overlapping mechanisms of lncRNA and expanded microsatellite RNA. Wiley Interdiscip Rev RNA.

[34] Kearse MG, Green KM, Krans A, Rodriguez CM, Linsalata AE, Goldstrohm AC, Todd PK (2016). CGG repeat-associated non-AUG translation utilizes a cap-dependent scanning mechanism of initiation to produce toxic proteins. Mol Cell.

[35] Kedersha N, Stoecklin G, Ayodele M, Yacono P, Lykke-Andersen J, Fritzler MJ, Scheuner D, Kaufman RJ, Golan DE, Anderson P (2005). Stress granules and processing bodies are dynamically linked sites of mRNP remodeling. J Cell Biol.

[36] Khateb S, Weisman-Shomer P, Hershco-Shani I, Ludwig AL, Fry M (2007). The tetraplex (CGG)n destabilizing proteins hnRNP A2 and CBF-A enhance the in vivo translation of fragile X premutation mRNA. Nucleic Acids Res.

[37] Kozlowski P, de Mezer M, Krzyzosiak WJ (2010). Trinucleotide repeats in human genome and exome. Nucleic Acids Res.

[38] Krans A, Skariah G, Zhang Y, Bayly B, Todd PK (2019). Neuropathology of RAN translation proteins in fragile X-associated tremor/ataxia syndrome. Acta Neuropathol Commun.

[39] La Spada AR, Wilson EM, Lubahn DB, Harding AE, Fischbeck KH (1991). Androgen receptor gene mutations in X-linked spinal and bulbar muscular atrophy. Nature.

[40] Lee YB, Chen HJ, Peres JN, Gomez-Deza J, Attig J, Stalekar M, Troakes C, Nishimura AL, Scotter EL, Vance C, Adachi Y, Sardone V, Miller JW, Smith BN, Gallo JM, Ule J, Hirth F, Rogelj B, Houart C, Shaw CE (2013). Hexanucleotide repeats in ALS/FTD form length-dependent RNA foci, sequester RNA binding proteins, and are neurotoxic. Cell Rep.

[41] Li YR, King OD, Shorter J, Gitler AD (2013). Stress granules as crucibles of ALS pathogenesis. J Cell Biol.

[42] Liu J, Hu J, Ludlow AT, Pham JT, Shay JW, Rothstein JD, Corey DR (2017). c9orf72 disease-related foci are each composed of one mutant expanded repeat RNA. Cell Chem Biol.

[43] Mahboubi H, Seganathy E, Kong D, Stochaj U (2013). Identification of Novel Stress Granule Components That Are Involved in Nuclear Transport. PLoS ONE.

[44] Mankodi A, Logigian E, Callahan L, McClain C, White R, Henderson D, Krym M, Thornton CA (2000). Myotonic dystrophy in transgenic mice expressing an expanded CUG repeat. Science.

[45] Mankodi A, Urbinati CR, Yuan QP, Moxley RT, Sansone V, Krym M, Henderson D, Schalling M, Swanson MS, Thornton CA (2001). Muscleblind localizes to nuclear foci of aberrant RNA in myotonic dystrophy types 1 and 2. Hum Mol Genet.

[46] Mehta AR, Selvaraj BT, Barton SK, McDade K, Abrahams S, Chandran S, Smith C, Gregory JM (2020) Improved detection of RNA foci in C9orf72 amyotrophic lateral sclerosis post-mortem tissue using BaseScope shows a lack of association with cognitive dysfunction. Brain Commun 2: fcaa009 10.1093/braincomms/fcaa009 PMC7099934 32226938

[47] Metz A, Soret J, Vourc'h C, Tazi J, Jolly C (2004). A key role for stress-induced satellite III transcripts in the relocalization of splicing factors into nuclear stress granules. J Cell Sci.

[48] Miller JW, Urbinati CR, Teng-Umnuay P, Stenberg MG, Byrne BJ, Thornton CA, Swanson MS (2000). Recruitment of human muscleblind proteins to (CUG)(n) expansions associated with myotonic dystrophy. EMBO J.

[49] Mizielinska S, Lashley T, Norona FE, Clayton EL, Ridler CE, Fratta P, Isaacs AM (2013). C9orf72 frontotemporal lobar degeneration is characterised by frequent neuronal sense and antisense RNA foci. Acta Neuropathol.

[50] Nussbacher JK, Tabet R, Yeo GW, Lagier-Tourenne C (2019). Disruption of RNA Metabolism in Neurological Diseases and Emerging Therapeutic Interventions. Neuron.

[51] Orr HT, Zoghbi HY (2007). Trinucleotide repeat disorders. Annu Rev Neurosci.

[52] Patterson JR, Wood MP, Schisa JA (2011). Assembly of RNP granules in stressed and aging oocytes requires nucleoporins and is coordinated with nuclear membrane blebbing. Dev Biol.

[53] Paulson HL, Perez MK, Trottier Y, Trojanowski JQ, Subramony SH, Das SS, Vig P, Mandel JL, Fischbeck KH, Pittman RN (1997). Intranuclear inclusions of expanded polyglutamine protein in spinocerebellar ataxia type 3. Neuron.

[54] Reddy K, Schmidt MH, Geist JM, Thakkar NP, Panigrahi GB, Wang YH, Pearson CE (2014). Processing of double-R-loops in (CAG). (CTG) and C9orf72 (GGGGCC). (GGCCCC) repeats causes instability. Nucleic Acids Res.

[55] Reddy K, Tam M, Bowater RP, Barber M, Tomlinson M, Nichol Edamura K, Wang YH, Pearson CE (2011). Determinants of R-loop formation at convergent bidirectionally transcribed trinucleotide repeats. Nucleic Acids Res.

[56] A. E. Renton, E. Majounie, A. Waite, J. Simon-Sanchez, S. Rollinson, J. R. Gibbs, J. C. Schymick, H. Laaksovirta, J. C. van Swieten, L. Myllykangas, H. Kalimo, A. Paetau, Y. Abramzon, A. M. Remes, A. Kaganovich, S. W. Scholz, J. Duckworth, J. Ding, D. W. Harmer, D. G. Hernandez, J. O. Johnson, K. Mok, M. Ryten, D. Trabzuni, R. J. Guerreiro, R. W. Orrell, J. Neal, A. Murray, J. Pearson, I. E. Jansen, D. Sondervan, H. Seelaar, D. Blake, K. Young, N. Halliwell, J. B. Callister, G. Toulson, A. Richardson, A. Gerhard, J. Snowden, D. Mann, D. Neary, M. A. Nalls, T. Peuralinna, L. Jansson, V. M. Isoviita, A. L. Kaivorinne, M. Holtta-Vuori, E. Ikonen, R. Sulkava, M. Benatar, J. Wuu, A. Chio, G. Restagno, G. Borghero, M. Sabatelli, Italsgen Consortium, D. Heckerman, E. Rogaeva, L. Zinman, J. D. Rothstein, M. Sendtner, C. Drepper, E. E. Eichler, C. Alkan, Z. Abdullaev, S. D. Pack, A. Dutra, E. Pak, J. Hardy, A. Singleton, N. M. Williams, P. Heutink, S. Pickering-Brown, H. R. Morris, P. J. Tienari, and B. J. Traynor (2011). A hexanucleotide repeat expansion in C9ORF72 is the cause of chromosome 9p21-linked ALS-FTD. Neuron.

[57] Rodriguez CM, Todd PK (2019). New pathologic mechanisms in nucleotide repeat expansion disorders. Neurobiol Dis.

[58] Rodriguez CM, Wright SE, Kearse MG, Haenfler JM, Flores BN, Liu Y, Ifrim MF, Glineburg MR, Krans A, Jafar-Nejad P, Sutton MA, Bassell GJ, Parent JM, Rigo F, Barmada SJ, Todd PK (2020). A native function for RAN translation and CGG repeats in regulating fragile X protein synthesis. Nat Neurosci.

[59] Rossi S, Serrano A, Gerbino V, Giorgi A, Di Francesco L, Nencini M, Bozzo F, Schinina ME, Bagni C, Cestra G, Carri MT, Achsel T, Cozzolino M (2015). Nuclear accumulation of mRNAs underlies G4C2-repeat-induced translational repression in a cellular model of C9orf72 ALS. J Cell Sci.

[60] Saxonov S, Berg P, Brutlag DL (2006). A genome-wide analysis of CpG dinucleotides in the human genome distinguishes two distinct classes of promoters. Proc Natl Acad Sci U S A.

[61] Sellier C, Freyermuth F, Tabet R, Tran T, He F, Ruffenach F, Alunni V, Moine H, Thibault C, Page A, Tassone F, Willemsen R, Disney MD, Hagerman PJ, Todd PK, Charlet-Berguerand N (2013). Sequestration of DROSHA and DGCR8 by expanded CGG RNA repeats alters microRNA processing in fragile X-associated tremor/ataxia syndrome. Cell Rep.

[62] Sellier C, Rau F, Liu Y, Tassone F, Hukema RK, Gattoni R, Schneider A, Richard S, Willemsen R, Elliott DJ, Hagerman PJ, Charlet-Berguerand N (2010). Sam68 sequestration and partial loss of function are associated with splicing alterations in FXTAS patients. EMBO J.

[63] Shah S, Lubeck E, Schwarzkopf M, He TF, Greenbaum A, Sohn CH, Lignell A, Choi HM, Gradinaru V, Pierce NA, Cai L (2016). Single-molecule RNA detection at depth by hybridization chain reaction and tissue hydrogel embedding and clearing. Development.

[64] Sofola OA, Jin P, Qin Y, Duan R, Liu H, de Haro M, Nelson DL, Botas J (2007). RNA-binding proteins hnRNP A2/B1 and CUGBP1 suppress fragile X CGG premutation repeat-induced neurodegeneration in a Drosophila model of FXTAS. Neuron.

[65] Sonobe Y, Ghadge G, Masaki K, Sendoel A, Fuchs E, Roos RP (2018). Translation of dipeptide repeat proteins from the C9ORF72 expanded repeat is associated with cellular stress. Neurobiol Dis.

[66] Spille JH, Hecht M, Grube V, Cho WK, Lee C, Cisse II (2019). A CRISPR/Cas9 platform for MS2-labelling of single mRNA in live stem cells. Methods.

[67] Sulovari, A., R. Li, P. A. Audano, D. Porubsky, M. R. Vollger, G. A. Logsdon, Consortium Human Genome Structural Variation, W. C. Warren, A. A. Pollen, M. J. P. Chaisson, and E. E. Eichler (2019). Human-specific tandem repeat expansion and differential gene expression during primate evolution. Proc Natl Acad Sci U S A.

[68] Swinnen B, Robberecht W, Van Den Bosch L (2020). RNA toxicity in non-coding repeat expansion disorders. EMBO J.

[69] Tantale K, Mueller F, Kozulic-Pirher A, Lesne A, Victor JM, Robert MC, Capozi S, Chouaib R, Backer V, Mateos-Langerak J, Darzacq X, Zimmer C, Basyuk E, Bertrand E (2016). A single-molecule view of transcription reveals convoys of RNA polymerases and multi-scale bursting. Nat Commun.

[70] Tassone F, Iwahashi C, Hagerman PJ (2004). FMR1 RNA within the intranuclear inclusions of fragile X-associated tremor/ataxia syndrome (FXTAS). RNA Biol.

[71] Todd PK, Oh SY, Krans A, He F, Sellier C, Frazer M, Renoux AJ, Chen KC, Scaglione KM, Basrur V, Elenitoba-Johnson K, Vonsattel JP, Louis ED, Sutton MA, Taylor JP, Mills RE, Charlet-Berguerand N, Paulson HL (2013). CGG repeat-associated translation mediates neurodegeneration in fragile X tremor ataxia syndrome. Neuron.

[72] Trottier Y, Lutz Y, Stevanin G, Imbert G, Devys D, Cancel G, Saudou F, Weber C, David G, Tora L (1995). Polyglutamine expansion as a pathological epitope in Huntington's disease and four dominant cerebellar ataxias. Nature.

[73] Tuduri S, Crabbe L, Conti C, Tourriere H, Holtgreve-Grez H, Jauch A, Pantesco V, De Vos J, Thomas A, Theillet C, Pommier Y, Tazi J, Coquelle A, Pasero P (2009). Topoisomerase I suppresses genomic instability by preventing interference between replication and transcription. Nat Cell Biol.

[74] Udan-Johns M, Bengoechea R, Bell S, Shao J, Diamond MI, True HL, Weihl CC, Baloh RH (2014). Prion-like nuclear aggregation of TDP-43 during heat shock is regulated by HSP40/70 chaperones. Hum Mol Genet.

[75] Urbanek MO, Krzyzosiak WJ (2016). RNA FISH for detecting expanded repeats in human diseases. Methods.

[76] Urbanek MO, Michalak M, Krzyzosiak WJ (2017). 2D and 3D FISH of expanded repeat RNAs in human lymphoblasts. Methods.

[77] Verma AK, Khan E, Mishra SK, Jain N, Kumar A (2019). Piperine Modulates protein mediated toxicity in fragile x-associated tremor/ataxia syndrome through interacting expanded CGG Repeat (r(CGG)(exp)) RNA. ACS Chem Neurosci.

[78] Wang C, Duan Y, Duan G, Wang Q, Zhang K, Deng X, Qian B, Gu J, Ma Z, Zhang S, Guo L, Liu C, Fang Y (2020). Stress induces dynamic, cytotoxicity-antagonizing TDP-43 nuclear bodies via paraspeckle LncRNA NEAT1-mediated liquid–liquid phase separation. Mol Cell.

[79] Weskamp K, Tank EM, Miguez R, McBride JP, Gomez NB, White M, Lin Z, Gonzalez CM, Serio A, Sreedharan J, Barmada SJ (2020). Shortened TDP43 isoforms upregulated by neuronal hyperactivity drive TDP43 pathology in ALS. J Clin Invest.

[80] Westergard T, McAvoy K, Russell K, Wen X, Pang Y, Morris B, Pasinelli P, Trotti D, Haeusler A (2019) 'Repeat-associated non-AUG translation in C9orf72-ALS/FTD is driven by neuronal excitation and stress. EMBO Mol Med 11 10.15252/emmm.201809423 PMC6365928 30617154

[81] Yamashita R, Suzuki Y, Sugano S, Nakai K (2005). Genome-wide analysis reveals strong correlation between CpG islands with nearby transcription start sites of genes and their tissue specificity. Gene.

[82] Young AP, Jackson DJ, Wyeth RC (2020). A technical review and guide to RNA fluorescence in situ hybridization. PeerJ.

[83] Yu H, Lu S, Gasior K, Singh D, Vazquez-Sanchez S, Tapia O, Toprani D, Beccari MS, Yates JR, Da Cruz S, Newby JM, Lafarga M, Gladfelter AS, Villa E, Cleveland DW (2020). HSP70 chaperones RNA-free TDP-43 into anisotropic intranuclear liquid spherical shells.

[84] Zhang K, Daigle JG, Cunningham KM, Coyne AN, Ruan K, Grima JC, Bowen KE, Wadhwa H, Yang P, Rigo F, Taylor JP, Gitler AD, Rothstein JD, Lloyd TE (2018). Stress granule assembly disrupts nucleocytoplasmic transport. Cell.

[85] Zhang K, Donnelly CJ, Haeusler AR, Grima JC, Machamer JB, Steinwald P, Daley EL, Miller SJ, Cunningham KM, Vidensky S, Gupta S, Thomas MA, Hong I, Chiu SL, Huganir RL, Ostrow LW, Matunis MJ, Wang J, Sattler R, Lloyd TE, Rothstein JD (2015). The C9orf72 repeat expansion disrupts nucleocytoplasmic transport. Nature.

[86] Zu T, Gibbens B, Doty NS, Gomes-Pereira M, Huguet A, Stone MD, Margolis J, Peterson M, Markowski TW, Ingram MA, Nan Z, Forster C, Low WC, Schoser B, Somia NV, Clark HB, Schmechel S, Bitterman PB, Gourdon G, Swanson MS, Moseley M, Ranum LP (2011). Non-ATG-initiated translation directed by microsatellite expansions. Proc Natl Acad Sci U S A.

