# Supplementary material for: Enhanced detection of expanded repeat mRNA foci with hybridization chain reaction
Source: Acta Neuropathol Commun. 2021 Apr 23;9:73. doi: 10.1186/s40478-021-01169-8 (PMC8063431; doi:10.1186/s40478-021-01169-8)
Supplement: Supplementary file 1 — Additional file 1. Supplemental Figures. [file 40478_2021_1169_MOESM1_ESM.pdf]

Supplemental Data:

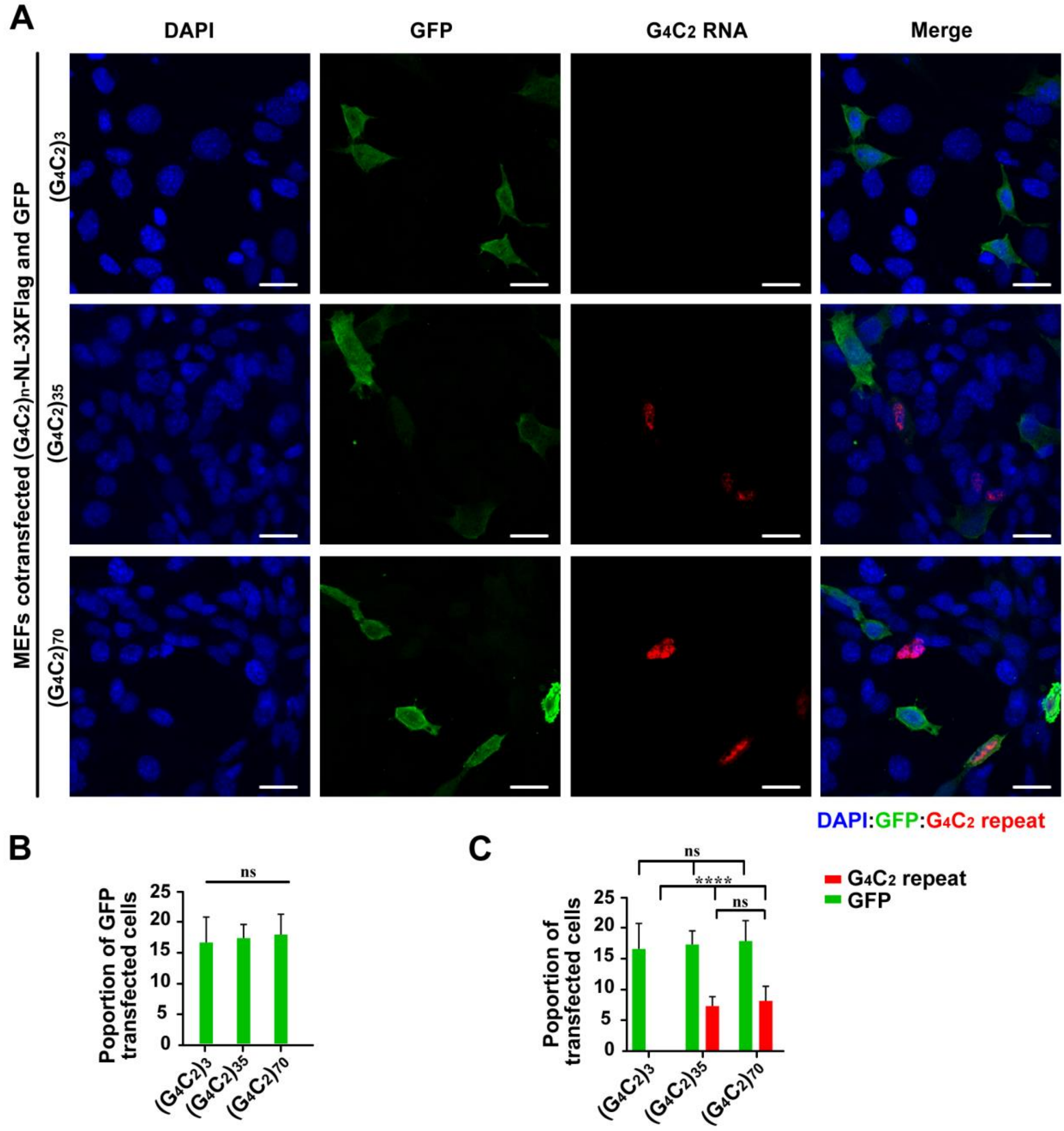

Supplemental Figure 1. Transfection efficiencies for MEFs co-transfected with GFP and G_4C_2 repeats A) R-HCR-ICC of MEFs co-transfected with GFP and G_4C_2 repeat constructs with increasing repeat size. **B-C)** Quantification of cells with detectable GFP and G_4C_2 repeat RNA foci signal in total cells. $(G_4C_2)_3$: N=313; $(G_4C_2)_{35}$ N=575; $(G_4C_2)_{70}$ N=514. Statistics: Chi-square test and Fisher's exact test **(B and C)**. ****p<0.0001, ns: not significant. Scale bar=50 μm in **A**.

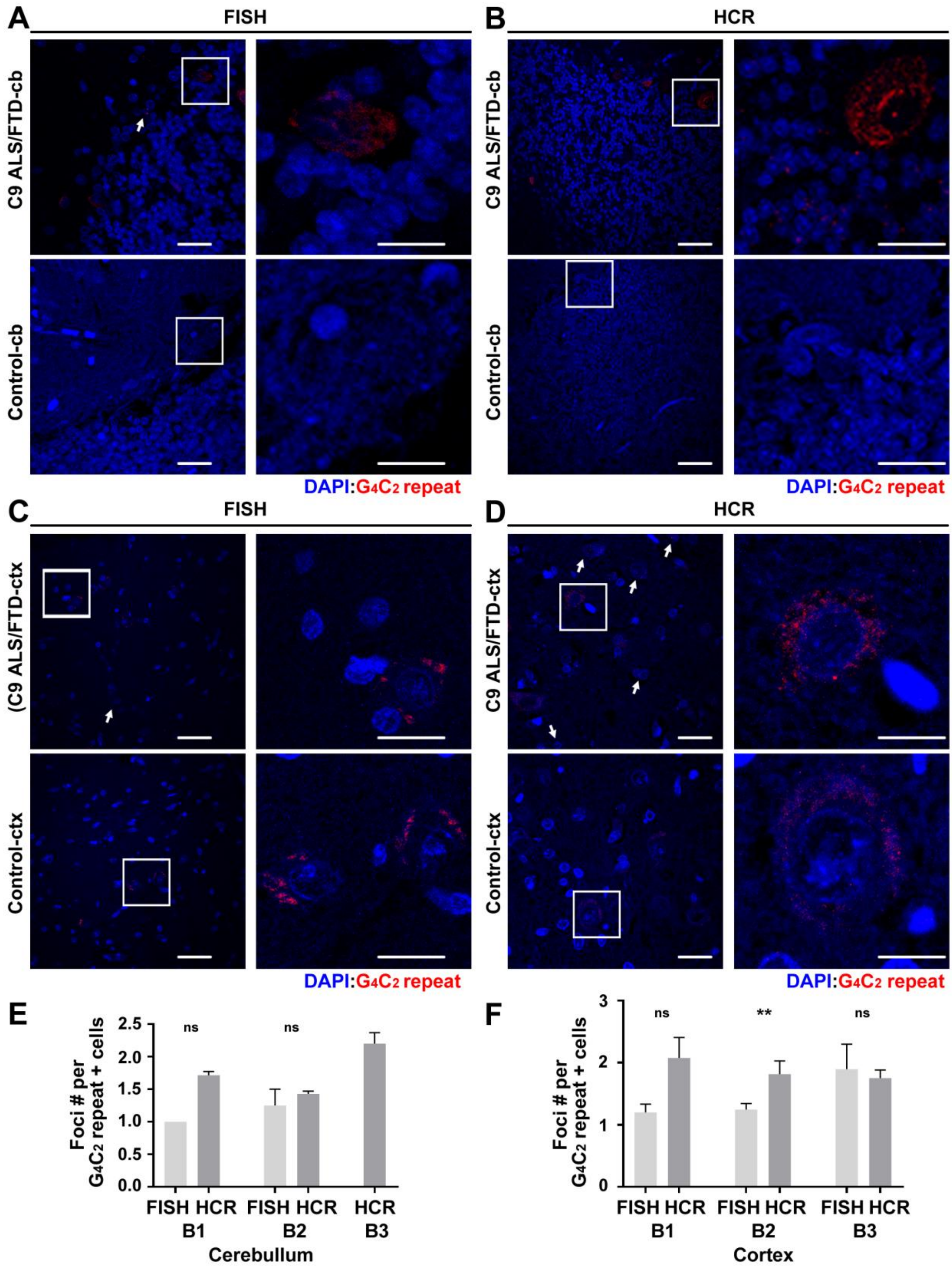

Supplemental Figure 2.

Supplemental Figure 2 legend. Diffuse R-HCR and FISH staining in *C9orf72* ALS-FTD patient brains. A-D) FISH **(A, C)** and R-HCR **(B, D)** of *C9orf72* ALS-FTD patient and control cerebellum **(A, B)** and frontal cortex **(C, D)**. White arrows indicate RNA foci positive cells in some images. Higher magnification images of the boxed areas are shown on the right. **E-F)** Quantification of RNA foci number per RNA foci + cells± SEM in cerebellum **(E)** and cortex **(F)**. Statistics: unpaired t test for E and F. ***p*<0.01, ns: not significant. Scale bar=50μm in left for A-D; 20μm in right for A-D.

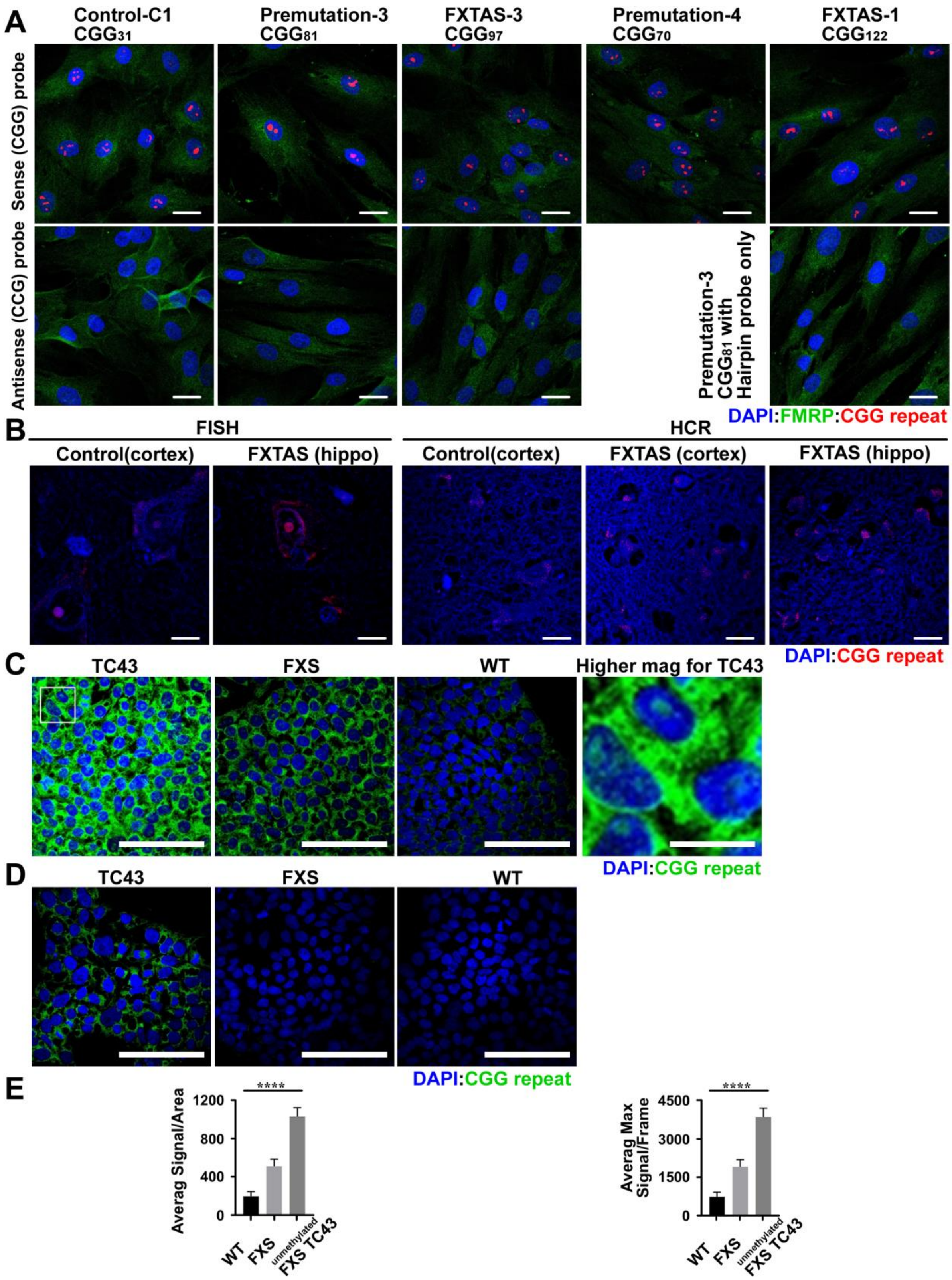

Supplemental Figure 3.

Supplemental Figure 3 legend: Endogenous CGG repeat is highly expressed in human cells. A) R-HCR of FXTAS, permutation carrier and control fibroblasts. **B)** FISH and R-HCR of FXTAS patient and control brains. **C-D)** CGG R-HCR of iPSCs/hESCs derived from an unmethylated CGG expansion carrier (TC-43; 270 CGG repeats, transcriptionally active), fully methylated embryonic cells (FXS, 800 CGG repeats, transcriptionally silent) and a control patient (30 CGG repeats). Panel **C** is without background subtraction, demonstrating signal in all cells. Panel **D** is after subtraction of signal detected in FXS line, revealing significant signal above background only in the actively transcribed expanded CGG repeat line. **E)** Quantification of CGG repeat RNA foci signal in each cell line of figure **(C)** *left:* average signal/frame, *right:* maximum signal/frame. One-way ANOVA (**E)**. n=15, ****p<0.0001. Scale bar=50μm in A; 20μm in B; 100μm in **C** and **D** except for the higher mag picture of **C** (20μm).
