# Supplementary material for: Enhanced detection of expanded repeat mRNA foci with hybridization chain reaction
Source: Acta Neuropathol Commun. 2021 Apr 23;9:73. doi: 10.1186/s40478-021-01169-8 (PMC8063431; doi:10.1186/s40478-021-01169-8)
Supplement: Supplementary file 2 — Additional file 2. Supplemental Tables. [file 40478_2021_1169_MOESM2_ESM.pdf]

Supplemental Table 1. Information for human derived samples.

Sample ID	Lab ID	Type	Diagnosis	CGG/G_4C_2 repeat NO.
C9-C1	499	fibroblasts	C9-ALS/FTD	G_4C_2=1180
C9-C2	733	fibroblasts	C9-ALS/FTD	G_4C_2=1100
C9-C3	953	fibroblasts	C9-ALS/FTD	G_4C_2=1230
Control-C1	C1	fibroblasts	Control	CGG=31
Control-C4	C4	fibroblasts	Control	CGG=22
C9-B1	exp198	Brain-cb	C9-ALS/FTD	Expanded (RP-PCR)
		Brain-cortex		
C9-B2	exp1233	Brain-cb	C9-ALS/FTD	Expanded (RP-PCR)
		Brain-cortex		
C9-B3	exp1350	Brain-cb	C9-ALS/FTD	Expanded (RP-PCR)
		Brain-cortex		
Control-B1	cont1033	Brain-cb	Control	n.d.
		Brain-cortex		
Control-B2	cont1533	Brain-cb	Control	n.d.
		Brain-cortex		
FXTAS-1	F1	fibroblast	FXTAS	CGG=122
FXTAS-3	F3	fibroblast	FXTAS	CGG=97
Premutation-3	P3	fibroblast	Premutation carrier	CGG=81
Premuation-4	P4	fibroblast	Premutation carrier	CGG=70
FXTAS-B1	1906	Brain-hippo	FXTAS	CGG=102
FXTAS-B2	T345	Brain-hippo	FXTAS	CGG=90
		Brain-cortex		
Control-B3	340	Brains-cortex	Control	n.d.
Control-B4	T357	Brains-cortex	Control	n.d.
C9-neuron	882	Neuron	C9-ALS/FTD	G_4C_2>44
Control-neuron	1021	Neuron	Control	G_4C_2=14
WT	wt4-6	iPSC	Control	CGG=30
TC43	TC43	iPSC	unmethylated FM	CGG=270
FXS	139-2	iPSC	FXS	CGG=~800

Supplemental table 2. LNA FISH probe and R-HCR probe sequences

Category	Product sequence 5’-3’
G_4C_2 repeat FISH LNA probe	/5TYE665/CCC+CGGC+CCC+GGC+CCCG+GCC+CCG+GCC+CCG+GCC+CCGG
CGG repeat FISH LNA probe	/5TYE665/CCG+CCGC+CGCCG+CCG+CCG+CCGCCG
G_4C_2 repeat initiator probe	GAGGAGGGCAGCAAACGGGAAGAGTCTTCCTTTACGATATTCCCCGGCCCCGGCCCCGGCCCCGGCCCCGGCCCCGGATATAGCATTCTTTCTTGAGGAGGGCAGCAAACGGGAAGAG
CGG repeat initiator probe	GAGGAGGGCAGCAAACGGGAAGAGTCTTCCTTTACGATATTCCGCCGCCGCCGCCGCCGCCGCCGATATAGCATTCTTTCTTGAGGAGGGCAGCAAACGGGAAGAG

+ represent the location for LNA

Supplemental table 3. Reporter Sequences

Reporter	Sequence
$(G_2C_4)_{47}$-NL-3xFlag	TAATACGACTCACTATAGGGAGACCCAAGCTGGCTAGCGTGTGTGTTTTTGTTTTTC<br>CCACCCTCTCTCCCCACTACTTGCTCTCACAGTACTCGCTGAGGGTGAACAAGAAA<br>AGACCTGATAAAGATTAACCAGAAGAAAACAAGGAGGGAAACAACCGCAGCCTGTA<br>GCAAGCTCTGGAACTCAGGatgAGTCGCGCTATCTAGACCGGCCCCGGCCCCG<br>GCCCCGGCCCCGGCCCCGGCCCCGGCCCCGGCCCCGGCCCCGGCCCCGGCCC<br>CGGCCCCGGCCCCGGCCCCGGCCCCGGCCCCGGCCCCGGCCCCGGCCCCGGC<br>CCCGGCCCCGGCCCCGGCCCCGGCCCCGGCCCCGGCCCCGGCCCCGGCCCCG<br>GCCCCGGCCCCGGCCCCGGCCCCGGCCCCTGCCCCGGCCCCGGCCCCGGCCC<br>CGGCCCCGGCCCCGGCCCCGGCCCCGGCCCCGGCCCCGGCCCCGGCCCCGGC<br>CCCGGCCCCTCTAGAAGCTTGGCAATCCGGTACTGTTGGTAAAGCCACCGGGGTC<br>TTCACACTCGAAGATTTCGTTGGGGACTGGCGACAGACAGCCGGCTACAACCTGG<br>ACCAAGTCCTTGAACAGGGAGGTGTGTCCAGTTTGTTTCAGAATCTCGGGGTGTCC<br>GTAACTCCGATCCAAAGGATTGTCCTGAGCGGTGAAAATGGGCTGAAGATCGACAT<br>CCATGTCATCATCCCGTATGAAGGTCTGAGCGGCGACCAAATGGGCCAGATCGAAA<br>AAATTTTTAAGGTGGTGTACCCTGTGGATGATCATCACTTTAAGGTGATCCTGCACTA<br>TGGCACACTGGTAATCGACGGGGTTACGCCGAACATGATCGACTATTTCGGACGGC<br>CGTATGAAGGCATCGCCGTGTTCGACGGCAAAAAGATCACTGTAACAGGGACCCTG<br>TGGAACGGCAACAAAATTATCGACGAGCGCCTGATCAACCCCGACGGCTCCCTGCT<br>GTTCCGAGTAACCATCAACGGAGTGACCGGCTGGCGGCTGTGCGAACGCATTCTGG<br>CGGACTACAAAGACCATGACGGTGATTATAAAGATCATGACATCGATTACAAGGATGA<br>CGATGACAAGTAAGGCCGCGACTCGAGAGGGCCC
FMRpolyG$_{100}$-3xF	GGATCCACTAGTCCAGTGTGGTGGAATTCGTTAACAGATCTGCTCAGCTCCGTTTCG<br>GTTTCACTTCCGGTGGAGGGCCGCCTCTGAGCGGGCGGCGGGCCGACGGCGAGC<br>GCGGGCGGCGGCGGTGACGGAGGCGCCGCTGCCAGGGGGCGTGCGGCAGCGCG<br>GCGGCGGCGGCGGCGGCGGCGGAGGCGGCGGCGGCGGCGGCGGCGGCGGCGG<br>CGGCGGCGGCGGCGGCGGCGGCGGCGGCGGCGGCGGCGGCGGCGGCGGCGGC<br>GGCGGCGGCGGCGGCGGCGGCGGCGGCGGCGGCGGCGGCGGCGGCGGCGGCG<br>GCGGCGGCGGCGGCGGCGGCGGCGGCGGCGGCGGCGGCGGCGGCGGCGGCGG<br>CGGCGGCGGCGGCGGCGGCGGCGGCGGCGGCGGCGGCGGCGGCGGCGGCGGC<br>GGCGGCGGCGGCGGCGGCGGCGGCGGCGGCGGCGGCGGCGGCTGGGCCTCGA<br>GCGCCCGCAGCCCACCTCTCGGGGGCGGGCTCCCGGCGCTAGCAGGGCTGAAGA<br>GAAGATGGAGGAGCTGGTGGTGGAAGTGCGGGGCTCCAATGGCGCTTTCTACAAGG<br>CATTTGAAAGCGGCCGCAGACTACAAAGACCATGACGGTGATTATAAAGATCATGACA<br>TCGACTACAAGGATGACGATGACAAGTAAGGGCCC
